# Toxin Induction or Inhibition of Transcription or Translation Posttreatment Increases Persistence to Fluoroquinolones

**DOI:** 10.1128/mBio.01983-21

**Published:** 2021-08-17

**Authors:** Annabel S. Lemma, Mark P. Brynildsen

**Affiliations:** a Department of Chemical and Biological Engineering, Princeton University, Princeton, New Jersey, USA; Mass General Hospital

**Keywords:** persister, TA module, nongrowing, stationary phase, recovery, persister

## Abstract

Toxin-antitoxin modules are widespread in prokaryotes, and the capacity of toxin accumulation to increase the tolerances of bacteria to antibiotics has been well documented. The conventional model for this functionality implies that an overabundance of toxin arrests bacterial growth, which inhibits processes targeted by antibiotics and thereby limits their corruption and the lethal damage that would ensue. Implicit in this model is that toxins exert their influence on antibiotic lethality before and/or during treatment, even though they are also present and functional after treatment concludes. Given recent evidence establishing that the period following antibiotic treatment (recovery) is important for the survival of nongrowing bacterial populations treated with fluoroquinolones (FQs), we assayed to what extent toxins influence bacterial survival during the recovery period. With both LdrD and MazF, toxins of type I and II systems, respectively, controlling accumulation to occur only after FQ treatment of nongrowing cultures resulted in significant increases in persisters. Further genetic investigation revealed important roles for homologous recombination and nucleotide excision repair machinery. Focusing on the wild type, we did not observe any SOS-induced toxin functioning in this manner; however, an analogous phenomenon was observed for wild-type Escherichia coli as well as uropathogenic E. coli (UPEC) when transcription or translation was inhibited during the post-FQ recovery period. Collectively, these data reveal the capacity of toxins to thwart FQ killing even after the treatment has concluded and show that FQ treatment of nongrowing bacteria can be rendered largely ineffective if bacteria cannot readily resume translation and growth at the conclusion of treatment.

## INTRODUCTION

Bacterial persisters exhibit increased tolerance to antibiotics in comparison to the majority of their kin, and their survival is not conferred by resistant mutations (1). Rather, persisters survive due to phenotypic reasons that continue to be elucidated (2–4). For this reason, genetics, environmental conditions, and epigenetic memory feature prominently in determining the abundances of persisters, because all influence phenotype (3). Importantly, experimental data have indicated that the majority of persisters in some populations are not multidrug tolerant, but rather, they contain different subsets of persisters that display tolerances to different antibiotics, with only a minority displaying uniform drug tolerance (5–9). For example, by using single and combination treatment with ampicillin (AMP) and/or ofloxacin (OFL), it was observed that persisters generated from a diauxic transition were largely distinct subpopulations, with approximately 10% exhibiting tolerance to both drugs (5). Just as many antibiotic resistance mechanisms display specificity to certain drug classes, it is expected that many mechanisms to achieve persistence will be drug specific (10). A prime example of this is the role of *recA* and *recB* in persistence to fluoroquinolones (FQs), which is not shared by other drug classes (11).

Genes encoding toxin-antitoxin (TA) modules are ubiquitous in bacterial species, and they have been linked to persistence since Moyed and Bertrand identified a high-persistence mutant that was later found to map to a TA module (12–14). TA systems consist of a toxin, which generally causes growth arrest by interfering with DNA replication, RNA stability, protein synthesis, or membrane integrity, and an antitoxin that prevents the toxin from participating in such growth-inhibitory exploits (15–19). The two major subclasses that encompass most of the identified TA modules in Escherichia coli are the type I and type II systems (20). In type I systems, the antitoxin is a small RNA that base-pairs to the mRNA of the toxin and prevents its expression (21). Type II TA modules, on the other hand, have a protein antitoxin that binds to the toxin protein to neutralize its effects or sequester it away from its target (22). Two prototypical examples of how toxins from type I and II systems influence persistence are TisB and HipA. TisB is induced during ciprofloxacin (CIP) treatment to prevent further damage, acting while the drug is still present (7), whereas HipA inhibits bacterial growth prior to and during antibiotic stress, allowing the bacteria to survive AMP treatment (2). Indeed, the conventional model of how toxins influence antibiotic tolerance is as follows: (i) accumulation of toxin in excess of its antitoxin allows toxins to act on their cellular targets to produce reversible growth inhibition; (ii) in the absence of growth, antibiotic-induced damage is reduced; and (iii) after antibiotic treatment has concluded, the eventual accumulation of the antitoxin or degradation of the toxin allows the bacteria to resume growth (2, 23). Experiments with toxin preexpression, such as with TisB or HipA (7, 24, 25), have lent support to this model. However, it is reasonable to assume that toxins expressed before or during antibiotic treatment remain following the conclusion of treatment. This raises the question of whether toxin activity following antibiotic treatment, rather than or in addition to before or during treatment, impacts bacterial survival.

In previous work, we had shown that the postantibiotic recovery period is critical to the survival of stationary-phase populations treated with FQs (26, 27). Here we use analogous stationary-phase populations to investigate the extent to which toxins impact survival following treatment with FQs. The toxins we used were LdrD, which is from a type I system, and MazF, which is from a type II system (22, 28). Using controlled expression of LdrD and MazF, we assessed whether toxin accumulation following FQ treatment altered bacterial survival. With both LdrD and MazF, we demonstrated that toxin induction in the post-FQ recovery period significantly increased persistence and that it depended on the function of RecA and UvrD. Further, we observed that toxin expression post-FQ treatment was as effective as expressing the toxin before and/or during antibiotic treatment for the nongrowing populations studied here. This suggested that the action of toxins after the conclusion of treatments is an underappreciated dimension of their function with regard to persistence. We then sought to assess whether this phenomenon played a role in wild-type persistence to FQs. To do this, we pursued two lines of inquiry. In the first, we reasoned that SOS-induced toxins were prime candidates for toxins that could impact survival following FQ treatment. However, a strain of E. coli devoid of all of the genes coding for known SOS-induced toxins (*tisB*, *symE*, *hokE*, *yafO*, *yafQ*, *dinQ*) (29) displayed persistence comparable to that of the wild type for stationary-phase cultures. In a second line of inquiry, we reasoned that emulating the actions of toxins through chemical means would result in the same outcome. Since MazF produces translational inhibition through mRNA degradation (30), we used rifampin (RIF) or chloramphenicol (CM) to chemically block transcription and translation after FQ treatment of stationary-phase cultures and, in both cases, observed significant increases in survival that approached 100%. Importantly, the ability of chemical inhibition to increase persistence in this manner paralleled that of post-FQ toxin induction with its dependence on RecA and UvrD. These observations recalibrate the landscape of how toxins can facilitate tolerance and persistence and suggest that any transcriptional or translational block following FQ treatment of nongrowing populations can foster bacterial survival.

## RESULTS

### Controlled expression of LdrD as a model persistence system.

**FIG 1:**
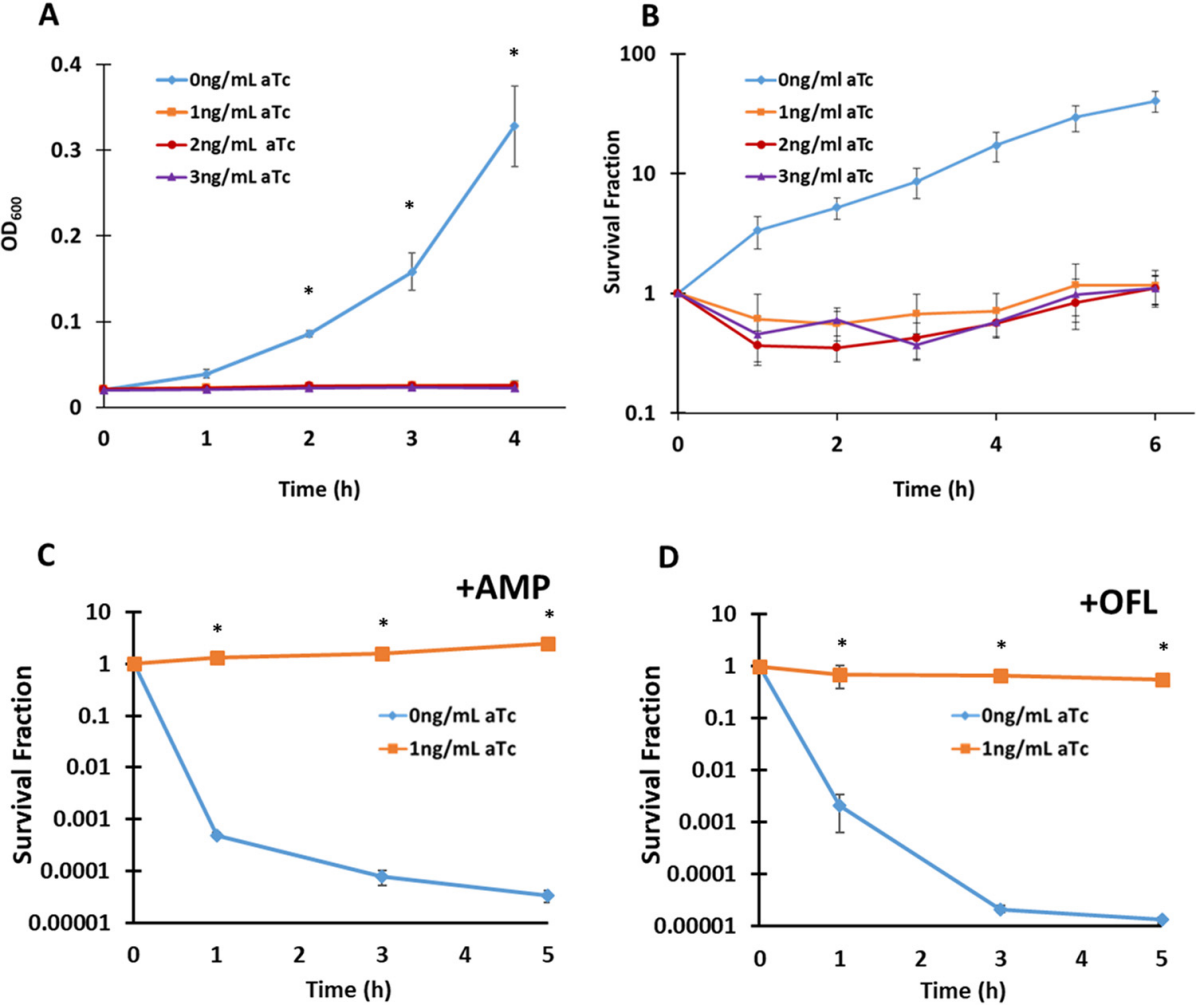
AL-*ldrD* can generate LdrD model persisters tolerant to AMP and OFL. (A) Exponential-phase cultures of AL-*ldrD* at an OD_600_ of 0.02 were treated with 1, 2, or 3 ng/ml aTc. OD_600_ was measured hourly to assess the effect of toxin induction on bacterial growth. All toxin-induced cells (1, 2, or 3 ng/ml) were growth inhibited starting at 1 h. (B) For culturability measurements, at *t* = 0, before the addition of aTc, and at 1, 2, 3, 4 5, and 6 h after the addition of the inducer, 500-μl samples were removed, washed three times, and plated. All toxin-induced samples exhibited a <5-fold decrease in the first 3 h before they regained culturability at 5 h. (C and D) Cells that were toxin induced for 5 h and controls were treated with OFL (5 μg/ml) or AMP (100 μg/ml). Five-hundred-microliter samples were taken at the designated time points and washed with PBS twice to reduce the antibiotic concentrations to below their MICs. Cells were then plated on LB agar and incubated for 16 h at 37°C. Toxin-induced AL-*ldrD* showed complete tolerance to AMP and OFL. Data points indicate the mean values of the results for at least three biological replicates with error bars indicating standard errors. An asterisk indicates statistical significance (*P* < 0.05) between toxin-induced and uninduced samples at the same time point.

Previously, our lab had constructed an E. coli strain with controlled expression of the toxin MazF and its antitoxin, MazE, which was used as a model persistence system (27, 30). MazF is an endoribonuclease and its TA system is a type II, which prompted us to develop another model persistence system based on a type I system, so that we could assess generality in this study. The type I toxin we selected was LdrD, whose antitoxin is *rdlD*, which interferes with translation of *ldrD* transcripts (28, 31, 32). Unlike the MazF model system, where expression of MazE quenches MazF toxicity (33), expression of *rdlD* cannot negate LdrD toxicity once it has been translated (31). Therefore, for the LdrD model system, *ldrD* and *rdlD* were genetically removed from the chromosome, and LdrD was reincorporated on the genome under the control of a tight anhydrotetracycline (aTc)-inducible promoter (see Materials and Methods and Fig. S1 in the supplemental material). In this construct, termed AL-*ldrD*, we demonstrated that LdrD expression could produce growth inhibition without a loss in culturability (Fig. 1A and B) and that those populations exhibited complete tolerance to AMP and OFL (Fig. 1C and D). Previous studies with toxins from type I systems, TisB and HokB, have discovered that their accumulations result in membrane depolarization and ATP leakage (34–36). To determine whether LdrD accumulation also resulted in membrane depolarization, we utilized the potential-sensitive DiBAC_4_(3) [bis-(1,3-dibarbituric acid)-trimethine oxanol] dye (37). The dye can enter depolarized cells, which produces increased fluorescence (37). A subpopulation of the LdrD-arrested cells exhibited increased fluorescence, whereas a nonarrested control (AL-empty strain) did not, which indicated that LdrD can facilitate membrane depolarization (Fig. S2A). However, with the majority of LdrD-arrested cells excluding the dye, these data also indicated that membrane depolarization was not required for the population-wide tolerance to fluoroquinolones that was observed (Fig. 1C). ATP measurements revealed that the LdrD-arrested populations exhibited increased extracellular ATP abundance per optical density at 600 nm (OD_600_) (Fig. S2B), which was similar to observations with HokB (35). When intracellular ATP was calculated from the difference of total culture ATP and extracellular ATP, a significant drop in ATP per OD_600_ was observed starting at the 1-h time point for AL-*ldrD* compared to AL-empty (Fig. S2C), which is similar to observations with TisB, where a 10% drop in ATP levels was recorded after 15 min of toxin induction (34). These data collectively show that the strain AL-*ldrD* can generate cells that are tolerant to different classes of antibiotics and that share some physiological traits with cells arrested by other toxins from type I systems.

10.1128/mBio.01983-21.1 FIG S1 Genetic construction of toxin-inducible strains and controls. Download FIG S1, TIF file, 0.1 MB. Copyright © 2021 Lemma and Brynildsen. 2021 Lemma and Brynildsen. https://creativecommons.org/licenses/by/4.0/ This content is distributed under the terms of the Creative Commons Attribution 4.0 International license.

10.1128/mBio.01983-21.2 FIG S2 LdrD accumulation results in membrane depolarization and ATP leakage, and the resulting LdrD model persisters experience DNA damage and utilize DNA repair mechanisms similar to those of wild-type persisters. (A) For cell depolarization assessment, AL-*ldrD* and AL-empty strains were grown to exponential phase in Gutnick glucose medium to an OD_600_ of 0.02. The culture was then treated with 3 ng/ml aTc. AL-*ldrD* strain was growth inhibited, whereas AL-empty exhibited exponential growth. At *t* = 3 h, samples were collected and treated with DiBAC_4_(3). AL-*ldrD* showed a spectral shift (right panel) indicative of cell depolarization. Controls where growth inhibition was achieved through inhibition of translation were treated with 50 μg/ml CM 1 h before DiBAC_4_(3) was introduced. The membrane depolarization-positive controls were treated with 100 μM CCCP for 15 min before DiBAC_4_(3) treatment. Histograms are representative of three biological replicates. (B and C) Total and extracellular ATP quantifications were carried out on exponential-phase cultures that were treated with or without 3 ng/ml aTc. Samples were collected at *t* = 0, 1, 2, and 3 h for ATP quantification. Induction of *ldrD* expression resulted in a significant increase in extracellular ATP. Intracellular ATP was calculated by subtracting the extracellular ATP concentration from the total ATP concentration of samples. There is a significant decrease in intracellular ATP in the toxin-inducible strain after 1 h of induction. An asterisk indicates statistical significance (*P* < 0.05) between AL-*ldrD* with aTc and AL-empty with aTc at the same time points. (D and E) Exponential-phase cultures of AL-*ldrD* strains devoid of DNA repair enzymes were treated with 3 ng/ml aTc for 5 h and then exposed to OFL for 5 h. Survival was monitored by taking samples at 0, 1, 3, and 5 h and washing the samples with PBS three times, followed by plating on LB agar. Strains with deletions of *recA* or *recB* exhibited declines in survival of approximately 4 to 5 orders of magnitude, whereas strains with *ruvA* and *uvrD* gene deletions exhibited declines in survival of approximately 1 order of magnitude compared to the parental strain. LdrD model persisters did not require *recN*, *recF*, *sulA*, *mutM*, *ung*, or *nfo* to tolerate OFL. Data points indicate the mean values of the results of at least three biological replicates with error bars indicating standard errors. An asterisk indicates statistical significance (*P* < 0.05) between a specific mutant and its parental strain (AL-*ldrD*) at the same time point. Download FIG S2, TIF file, 0.4 MB. Copyright © 2021 Lemma and Brynildsen. 2021 Lemma and Brynildsen. https://creativecommons.org/licenses/by/4.0/ This content is distributed under the terms of the Creative Commons Attribution 4.0 International license.

### LdrD persisters require DNA repair systems similar to the wild type to survive OFL treatment.

To assess to what extent LdrD persisters reflect wild-type persistence to FQs, we performed experiments with DNA repair mutants that have and have not been identified as important to wild-type FQ persisters (26). LdrD persister levels were significantly lower when *recA*, *recB*, *uvrD*, and *ruvA* were deleted, whereas loss of *nfo*, *mutM*, *ung*, *sulA*, *recF*, and *recN* did not reduce FQ persistence appreciably (Fig. S2D and E). These dependencies compare well to FQ persistence in stationary-phase wild-type populations where deletions of *recA*, *recB*, *uvrD*, and *ruvA* have been found to reduce persister levels, whereas deletion of *recF* had little impact (26). Both LdrD and MazF persisters require *recA*, *recB*, *uvrD*, and *ruvA* when challenged with FQ, whereas *nfo*, *mutM*, *ung*, *sulA*, *recF*, and *recN* were dispensable, which highlights a strong parallel between the two model persistence systems despite tolerance being achieved with mechanistically distinct toxins (27). The similarities between wild-type persisters and toxin model persisters with respect to the DNA repair enzymes they require under FQ treatment suggests that study of toxin-arrested cells could be useful for understanding FQ persistence in the wild type (26).

### Toxin induction after OFL treatment increases survival.

**FIG 2:**
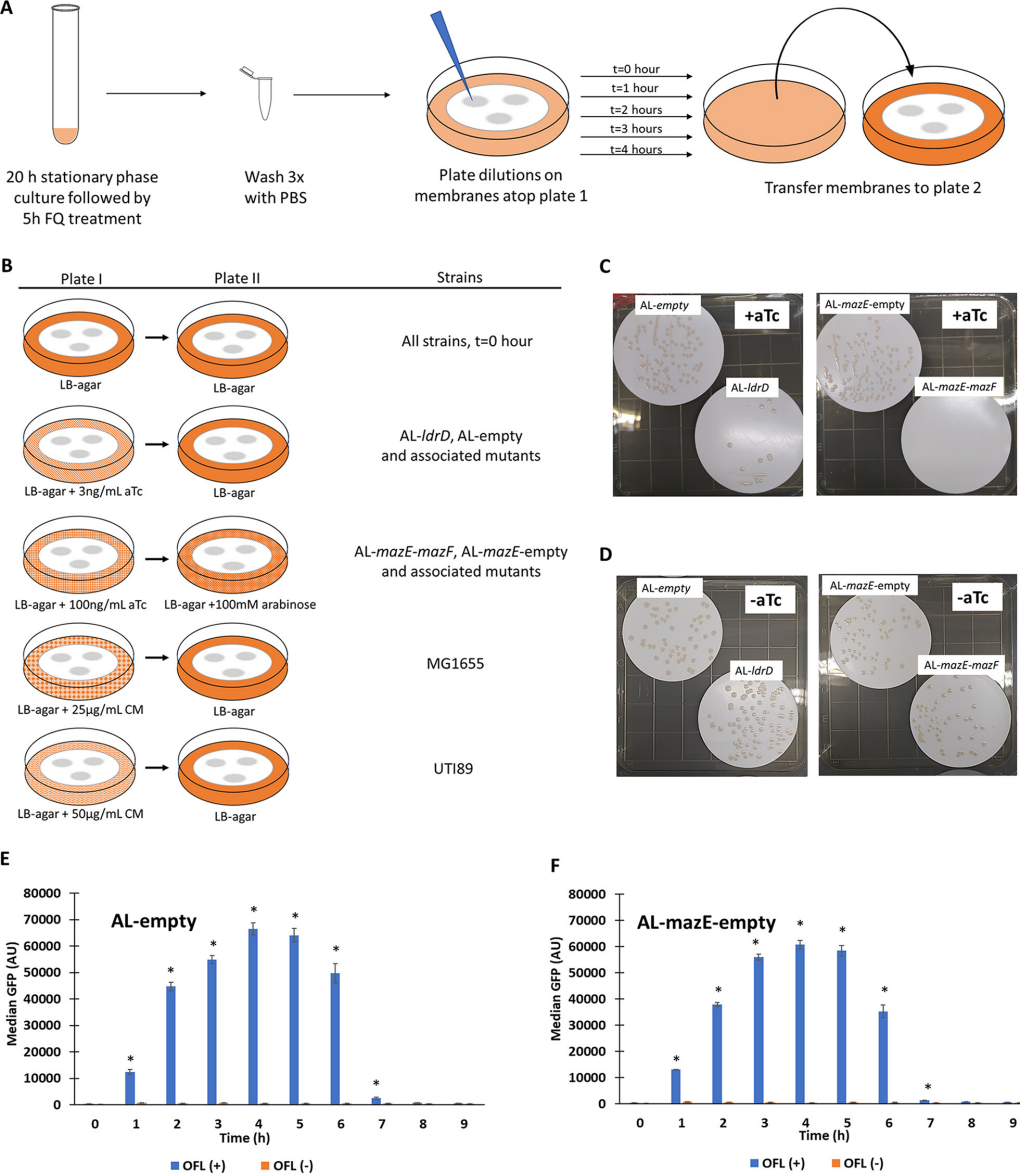
Experimental setup for recovery assays. (A) Cultures that had been grown to stationary phase in Gutnick medium (20 h) were treated with FQ for 5 h before they were washed with PBS three times and plated on Supor 200 membranes on top of LB agar plates with or without aTc or CM. At 0, 1, 2, 3, or 4 h, membranes were transferred to plate 2, where they were incubated at 37°C for 24 h, at which time the CFU were measured. (B) Details of plate 1 and plate 2 compositions for recovery assays performed. (C and D) Untreated, stationary-phase cultures of LdrD- and MazF- inducible strains and controls were plated on LB agar plates with or without aTc (3 ng/ml aTc for AL-*ldrD* and AL-empty; 100 ng/ml aTc for AL-*mazE*-*mazF* and AL-*mazE*-empty) and incubated at 37°C for 16 h. There was growth inhibition for the toxin-inducible strains on plates with aTc, whereas the control strains readily formed colonies. Comparable growth was observed between the controls and toxin-inducible strains on LB agar plates without aTc. (E and F) Average median fluorescence values were plotted for AL-empty and AL-*mazE*-empty strains containing an SOS reporter plasmid (pUA66 with P_recA_-*gfp*) during the recovery period after the completion of 5 h of OFL treatment. Maximum fluorescence values were observed at hour 4, and basal fluorescence values were reached at hour 8. An asterisk indicates statistical significance (*P* < 0.05) between treated and untreated samples.

In order to deconvolute the ability of toxins to facilitate FQ tolerance when present before, during, or after antibiotic treatment, we used the AL-*ldrD* and AL-*mazE*-*mazF* strains and their associated control strains, AL-empty and AL-*mazE*-empty, respectively (Fig. S1). In this assay, cells were grown to stationary phase in minimal medium in the absence of any inducer and then treated with OFL for 5 h (treatment period). FQ-treated cultures were then plated on LB agar with or without aTc for 1, 2, 3, or 4 h (recovery period) and then transferred to LB agar. Figure 2A and B provides a schematic of how this assay was conducted with the different strains and conditions investigated in this study. Experiments on untreated cultures demonstrated the ability of LdrD and MazF expression to inhibit growth on the aTc-containing LB agar plates (Fig. 2C and D). Experiments with an SOS fluorescent reporter strain indicated that the recovery period, which we defined as the time period that cells exhibited the impacts of FQ treatment, was 4 h or longer (Fig. 2E and F; Fig. S3).

10.1128/mBio.01983-21.3 FIG S3 SOS fluorescent reporter during the recovery period. AL-empty and AL-*mazE*-empty strains containing an SOS reporter plasmid (pUA66 with P_recA_-*gfp*) were treated with OFL for 5 h, washed in PBS, and diluted into recovery medium (LB). At *t* = 0 h before inoculation and at *t* = 1, 2, 3, 4, 5, 6, 7, 8, and 9 h, samples were collected for green fluorescent protein (GFP) fluorescence measurement. Fluorescence for OFL-treated samples increased during the recovery period until 4 h before it started to decline and reached basal levels at 8 h. Histograms are representative of three biological replicates. Download FIG S3, TIF file, 2.7 MB. Copyright © 2021 Lemma and Brynildsen. 2021 Lemma and Brynildsen. https://creativecommons.org/licenses/by/4.0/ This content is distributed under the terms of the Creative Commons Attribution 4.0 International license.

**FIG 3:**
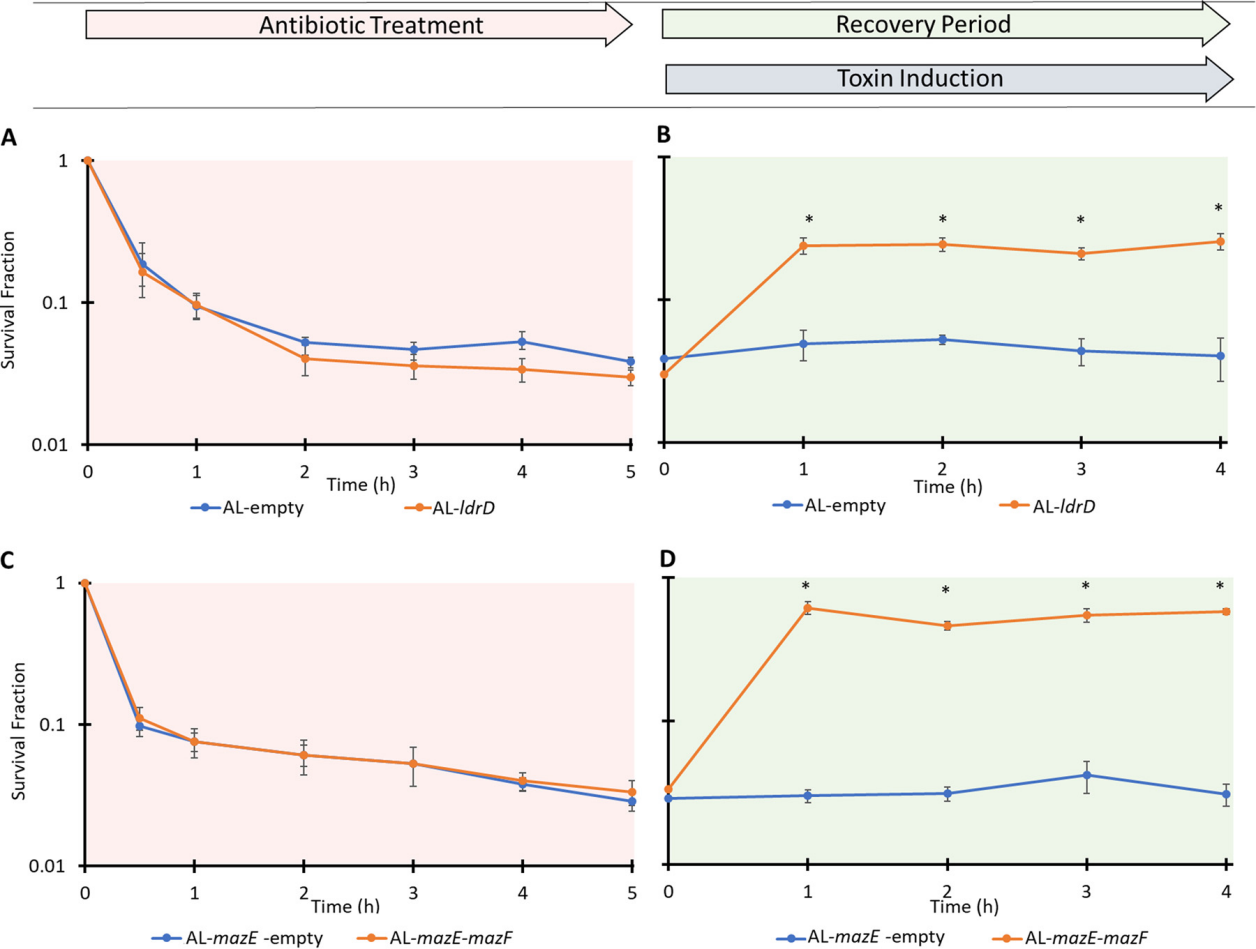
Post-OFL toxin induction rescues stationary-phase E. coli cells. (A) Stationary-phase AL-*ldrD* and AL-empty cultures grown in Gutnick minimal glucose medium were treated with OFL for 5 h. Comparable biphasic kill curves were observed for AL-*ldrD* and AL-empty samples. (B) After 5 h of OFL treatment, AL-*ldrD* and AL-empty samples were removed, washed, and plated onto filters placed on top of LB agar with 3 ng/ml aTc. At 1, 2, 3, or 4 h, the filters were transferred from LB agar with aTc to LB agar. (C) Stationary-phase AL-*mazE*-*mazF* and AL-*mazE*-empty cultures grown in Gutnick minimal glucose medium exhibited comparable biphasic kill curves under treatment with OFL. (D) The recovery assay was performed on 5-h OFL-treated AL-*mazE*-*mazF* and AL-*mazE*-empty cultures by plating them on LB agar plates containing 100 ng/ml aTc for 1, 2, 3, or 4 h before being transferred to LB agar plates with 100 mM arabinose. Diagrams of these procedures are shown in Fig. 2A and B. Survival fractions were calculated based on the CFU/ml measurements in cultures just prior to OFL treatment. Data points indicate the mean values of the results of at least three biological replicates with error bars indicating standard errors. In panels B and D, an asterisk indicates statistical significance (*P* < 0.05) between time points and initial (*t* = 0) time points in those assays.

During the 5-h treatment period, comparable biphasic kill curves were observed for both AL-*ldrD* samples and AL-empty controls when immediately plated on LB agar following washes to remove OFL (Fig. 3A). However, if cultures were plated on LB agar with aTc after the treatment period and then transferred to LB agar after 1, 2, 3, or 4 h, the survival of the AL-empty strain remained the same, whereas AL-*ldrD* increased significantly and was approximately 8-fold higher at 1, 2, 3, and 4 h, leading to approximately 25% of cells tolerating treatment with 5 μg/ml OFL (Fig. 3B). This suggested that post-OFL expression of LdrD might increase persistence. When analogous experiments were conducted with strains that could express MazF and/or MazE (Fig. S1), similar kill curves were observed for both AL-*mazE*-empty and AL-*mazE*-*mazF* strains (Fig. 3C), but the survival of AL-*mazE*-empty was unchanged by 1, 2, 3, or 4 h of post-OFL plating on LB agar with aTc, whereas the survival of AL-*mazE*-*mazF* increased significantly and was approximately 15-fold higher at 1, 2, 3, and 4 h, which resulted in approximately 60% of the cells tolerating treatment (Fig. 3D). These data with a completely distinct TA system suggested that expression of toxins following treatment with OFL could significantly increase persistence in nongrowing populations. However, we note that the same phenomenon was not observed with cultures that were treated with OFL during exponential growth (Fig. S4A and B), which paralleled previous results regarding the ability of starvation to boost FQ persistence (27).

10.1128/mBio.01983-21.4 FIG S4 Toxin-assisted recovery is observed with different FQ treatments for stationary-phase cultures but not for exponential-phase cultures. Persistence and recovery assays were performed using 5 μg/ml of ofloxacin (OFL), 1 μg/ml of ciprofloxacin (CIP), or 5 μg/ml of moxifloxacin (MOX). (A) Exponential-phase AL-*ldrD* and AL-*mazE-mazF* strains and their controls (AL-empty, AL-*mazE*-empty) were treated with OFL for 5 h. A biphasic kill curve was observed for all samples. (B) For the recovery assay, cultures were plated on LB agar plates with or without aTc for 1, 2, 3, or 4 h (3 ng/ml aTc for AL-*ldrD* and AL-empty; 100 ng/ml for AL-*mazE*-*mazF* and AL-*mazE*-empty) before being transferred to LB agar plates with or without arabinose (100 mM arabinose for AL-*mazE*-*mazF* and AL-*mazE*-empty). There was no increase in cell survival during the recovery assay for exponential-phase cultures. (C and D) A persister assay revealed that treatment of stationary-phase cells with CIP results in an ∼10-fold loss in survival (C), whereas MOX treatment results in an ∼100-fold decrease in survival across all samples (D). (E) Toxin-assisted recovery for CIP-treated cells resulted in approximately a 3-fold increase for AL-*ldrD* and 5-fold increase for AL-*mazE-mazF.* Survival of control strains without toxins remained constant during the recovery assays. (F) Induction of toxins post-MOX treatment resulted in an ∼10-fold increase in survival for both AL-*ldrD* and AL-*mazE-mazF*. Survival of control strains without toxins remained constant during the recovery assays. Data points indicate the mean values of the results of at least three biological replicates ± standard errors. An asterisk indicates statistical significance (*P* < 0.05) with respect to the same sample at *t* = 0 h. Download FIG S4, TIF file, 0.3 MB. Copyright © 2021 Lemma and Brynildsen. 2021 Lemma and Brynildsen. https://creativecommons.org/licenses/by/4.0/ This content is distributed under the terms of the Creative Commons Attribution 4.0 International license.

**FIG 4:**
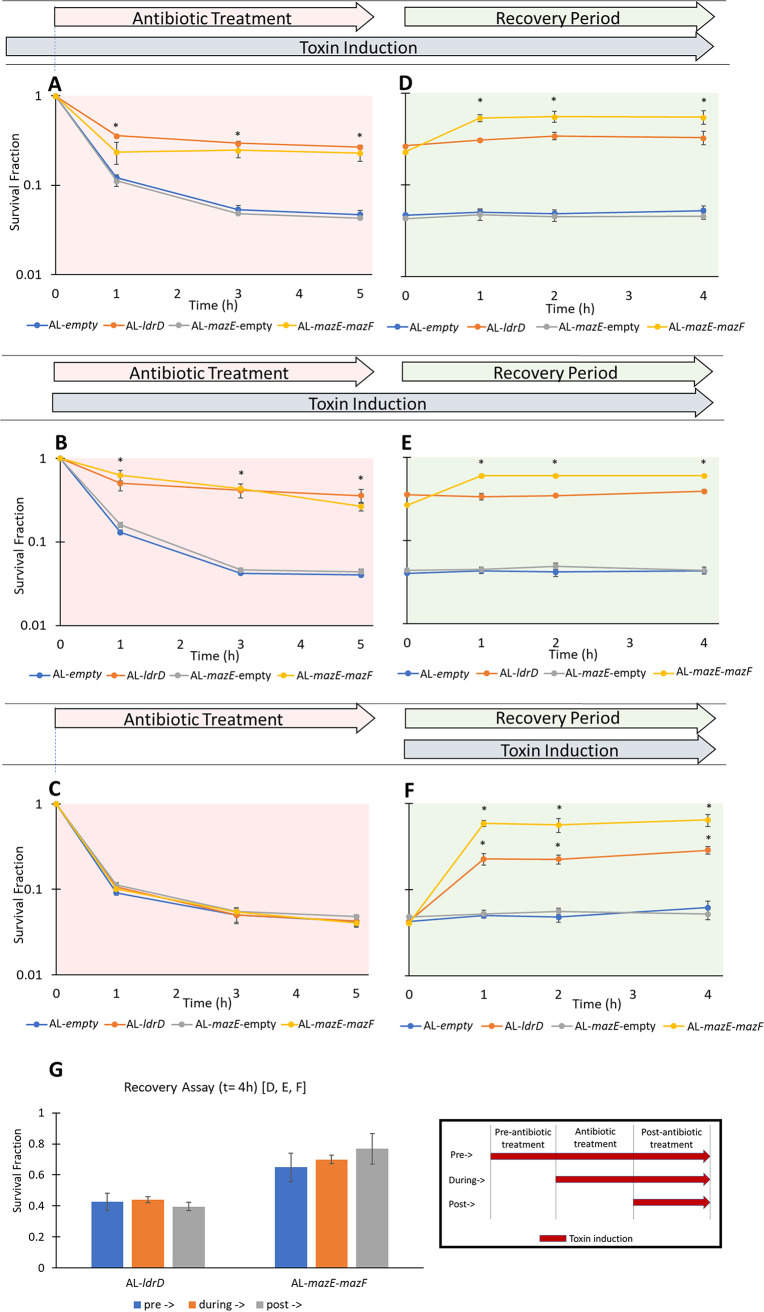
Starting toxin induction before and during OFL treatment results in survival levels similar to those with toxin induction only after treatment. With toxin induction before (pre-) and during antibiotic treatment, a majority of the cells survived without the need for extending toxin induction to the postantibiotic treatment stage. (A to C) MG1655 stationary-phase cultures were diluted 100-fold in spent medium, and 3 ng/ml aTc (LdrD) or 100 ng/ml aTc (MazF) was introduced at *t* = 18 h (pre-) or *t* = 20 h (during) of growth; experiments conducted on 100-fold-diluted stationary-phase cultures without toxin induction before or during antibiotic treatment are labeled “post-” above. (A and B) Induction of toxin starting 2 h before antibiotic treatment and extending through antibiotic treatment (pre-) (A) and induction of toxin starting during antibiotic treatment (during) (B) resulted in comparable 8-fold increases in survival for AL-*ldrD* and comparable 10-fold increases for AL-*mazE-mazF* relative to controls (AL-empty, AL-*mazE*-empty). (C) Samples where there was no toxin induction before or during antibiotic treatment exhibited the expected biphasic kill curve under OFL treatment. (D and E) Extending toxin induction after antibiotic treatment had concluded for “pre-“ and “during“ samples showed no change in survival for AL-*ldrD*, whereas there was a significant 3-fold increase for AL-*mazE-mazF*. Survival of control strains (AL-empty, AL-*mazE*-empty) was not altered by the inducer during the recovery phase of the assays. (F) Inducing toxins only after the conclusion of antibiotic treatment resulted in 8-fold increases in survival for AL-*ldrD* and 15-fold increases for AL-*mazE-mazF.* (G) Comparison of survival fractions at assay end points (*t* = 4 h in survival assay) from panels D to F. There was no significant difference in survival at *t* = 4 h of recovery between the three conditions in which toxin induction was started before, during, or after antibiotic treatment. The data points indicate the mean values of the results of at least three biological replicates ± standard error. For the persister assays, an asterisk indicates statistical significance (*P* < 0.05) with respect to control strains at the same time point. For the recovery assays, an asterisk indicates statistical significance (*P* < 0.05) with respect to *t* = 0 h of the same sample.

In order to see if the observed effect was generalizable to other FQs, we performed analogous assays with CIP and moxifloxacin (MOX). Similar to OFL, induction of toxins post-CIP or -MOX treatment of stationary-phase cultures resulted in significant increases in survival for AL-*ldrD* and AL-*mazE*-*mazF*, whereas the culturability of AL-empty and AL-*mazE*-empty were unchanged (Fig. S4C to F). When toxin induction was started before or during antibiotic treatment, survival at the end of persistence assays for the before- and during-treatment samples increased to levels similar to those observed when toxin was expressed only during recovery (Fig. 4A and B). When there was no toxin induction before or during antibiotic treatment, samples exhibited the expected biphasic kill curve (Fig. 4C). For those samples in which toxin induction was started before or during antibiotic treatment, extending toxin induction to the postantibiotic treatment stage elicited no change in survival for AL-*ldrD*, whereas there was an ∼3-fold increase for AL-*mazE-mazF* (Fig. 4D to F). There was no significant difference in the final cell survival levels during the recovery assay when toxin induction was started before, during, or after antibiotic treatment for both AL-*ldrD* and AL-*mazE-mazF* cells (Fig. 4G). These data demonstrated that toxin activity following the conclusion of FQ treatment was sufficient to achieve survival levels reached when toxins were present even before treatment began for nongrowing bacterial cultures.

### RecA is involved in, but not a requirement for, toxin-assisted rescue.

**FIG 5:**
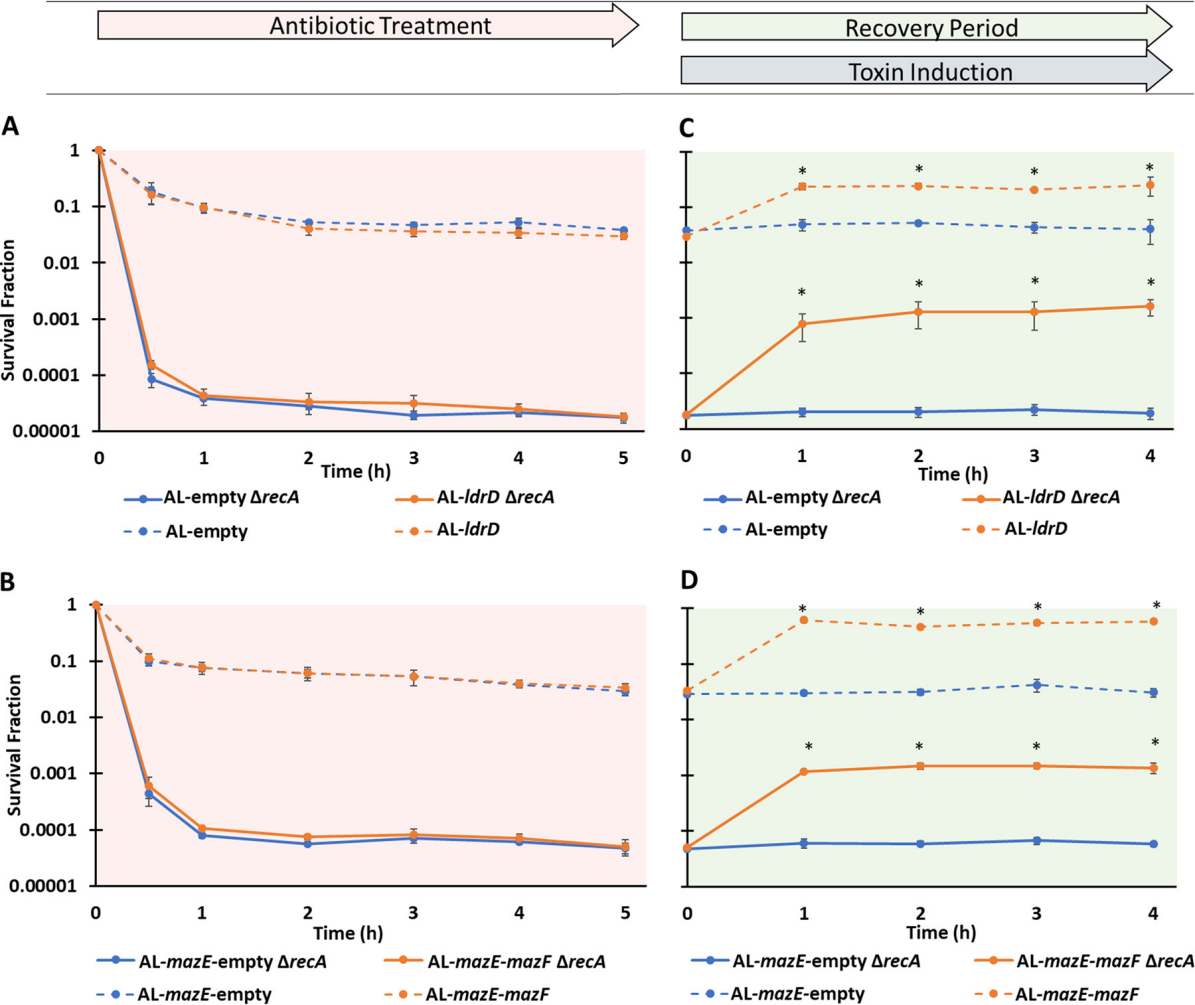
RecA is not required to observe toxin-assisted rescue. (A and B) Stationary-phase E. coli samples were treated with 5 μg/ml OFL for 5 h, and the numbers of CFU/ml were monitored. Strains without *recA* exhibited significant ∼10^3^-fold decreases in survival compared to wild-type cells under OFL treatment. (C and D) At 5 h post-OFL treatment, cells were washed and plated on filters placed on top of LB agar plates with or without aTc. At the indicated time points, filters were transferred to LB agar plates (AL-*ldrD*, AL-empty, and their mutants) or LB agar with 100 mM arabinose (AL-*mazE*-*mazF*, AL-*mazE*-empty, and their mutants). Data points indicate the mean values of the results of at least three biological replicates with error bars indicating standard errors. In panels C and D, an asterisk indicates statistical significance (*P* < 0.05) between time points and initial (*t* = 0) time points in those assays.

Previous work from our group demonstrated that following FQ treatment, the relative timing of DNA repair and growth resumption was important to the persistence of nongrowing bacterial populations (27). Allowing bacteria to recover from FQ treatment on medium that could not support growth increased survival up to ∼10-fold, as long as RecA, which is involved in homologous recombination and the SOS response, was present (27). In the absence of RecA, survival did not increase by starvation during the recovery period (27). Inspired by that work, we hypothesized that the post-FQ expression of toxins increased survival due to their ability to inhibit growth and thus allow DNA repair to function prior to growth resumption. To test this hypothesis, we deleted *recA* from the toxin-inducible strains and their respective controls. When those strains were subjected to OFL persistence assays, biphasic killing was observed and survival was far lower than that of the parental strains, which is consistent with previous work on Δ*recA* mutants (26, 38, 39) (Fig. 5A and B). When those cultures that had undergone 5 h of OFL treatment were plated on medium containing aTc for 1, 2, 3, or 4 h, survival was unchanged in the control strains that did not express toxins, whereas for those that expressed toxins, significant increases in survival were observed (∼100-fold for LdrD, ∼25-fold for MazF) (Fig. 5C and D). This result was unexpected and suggested that *recA* was not required to observe the post-FQ enhancement of survival by toxin expression. However, the absolute number of persisters after toxin-assisted rescue was ∼100-fold less in Δ*recA* strains than in their *recA*-containing counterparts (Fig. 5C and D; Fig. S5). These data indicated that although the fold change in survival enhancement was similar between Δ*recA* and *recA*-containing strains, ∼1 × 10^6^ cells per ml were rescued by toxin induction in Δ*recA* strains, whereas ∼1 × 10^8^ cells per ml were rescued in *recA*-containing strains (Fig. S5). This suggested that RecA was still needed by the majority of cells rescued through toxin induction post-FQ treatment, even though Δ*recA* strains exhibited toxin-assisted rescue. We considered that an additional DNA repair mechanism might be involved in the toxin-assisted rescue of Δ*recA* populations.

10.1128/mBio.01983-21.5 FIG S5 Absolute culturability levels for LdrD- and MazF-inducible strains and controls in Δ*recA* and parental strains. The numbers of CFU/ml reveal that the absolute number of persisters after toxin-assisted rescue was approximately 100-fold less in Δ*recA* strains than in their *recA*-containing counterparts in both the LdrD- and MazF-inducible strains. Download FIG S5, TIF file, 0.4 MB. Copyright © 2021 Lemma and Brynildsen. 2021 Lemma and Brynildsen. https://creativecommons.org/licenses/by/4.0/ This content is distributed under the terms of the Creative Commons Attribution 4.0 International license.

### UvrD is required for toxin-assisted rescue in Δ*recA* populations.

**FIG 6:**
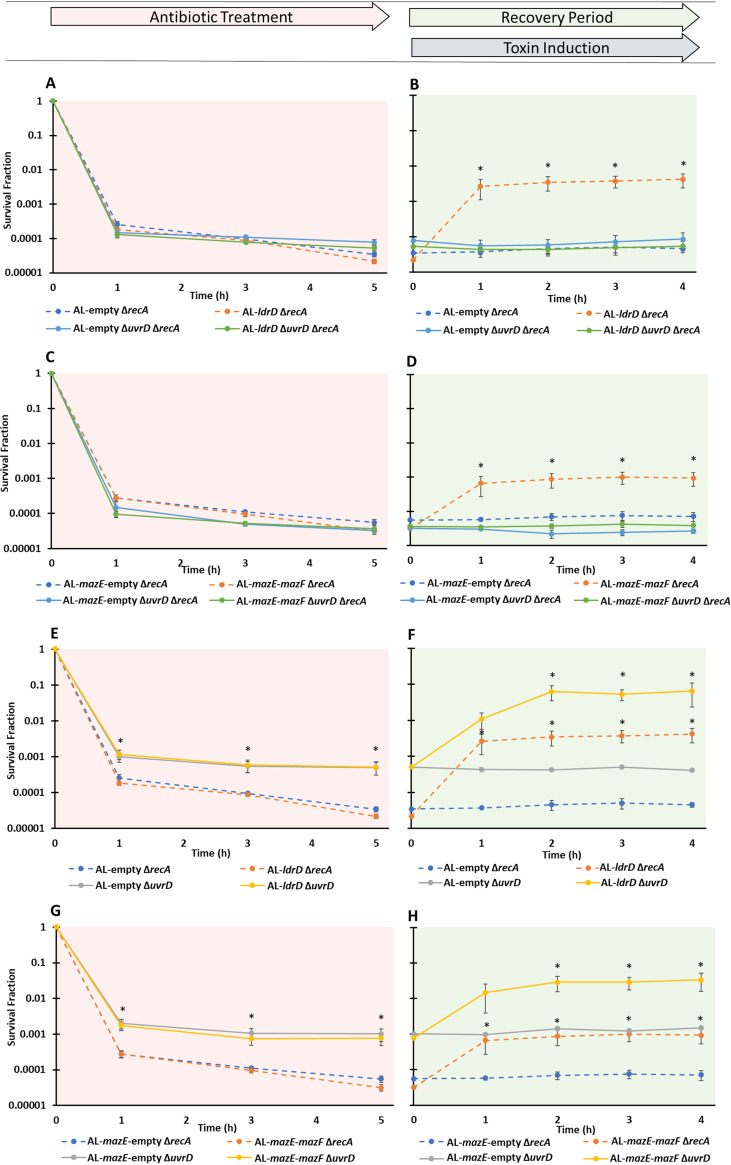
UvrD is required for toxin-assisted recovery only in the absence of *recA*. (A) OFL-treated (5 μg/ml) stationary-phase AL-*ldrD* Δ*uvrD* Δ*recA* and control strains showed survival curves comparable to that of the AL-*ldrD* Δ*recA* strain. (B) For the recovery assay, survival remained unchanged for the toxin-inducible AL-*ldrD* Δ*uvrD* Δ*recA* strain upon aTc induction. (C) AL-*mazE-mazF* and AL-*mazE*-empty strains harboring the Δ*uvrD* Δ*recA* deletions similarly exhibited survival curves comparable to those of their analogous Δ*recA* strains when undergoing 5-h OFL treatment. (D) During the recovery assay, there was no observed rescue for the AL-*mazE-mazF* Δ*uvrD* Δ*recA* strain with the induction of the toxin. (E) Stationary-phase AL-*ldrD* and AL-empty Δ*uvrD* strains showed ∼100-fold decreases in survival when treated with OFL in comparison to analogous strains without the deletion. (F) LdrD induction resulted in a significant 100-fold increase in survival for AL-*ldrD* Δ*uvrD*. (H) Similarly, MazF-inducible strains with the Δ*uvrD* deletion exhibited ∼100-fold decreases in survival under OFL treatment compared to AL-*mazE-mazF.* Toxin induction during recovery resulted in a significant ∼40-fold increase in survival for AL-*mazE*-*mazF* Δ*uvrD*. Exposure to aTc for all control strains did not significantly alter survival. Data points indicate the mean values of the results of at least three biological replicates, and error bars reflect standard errors. For the persister assays, an asterisk indicates statistical significance (*P* < 0.05) with respect to control strains at the same time point. For the recovery assays, an asterisk indicates statistical significance (*P* < 0.05) with respect to the same sample at *t* = 0 h.

To assess the involvement of other DNA repair mechanisms in toxin-assisted rescue of Δ*recA* strains, we introduced deletion mutations (Δ*uvrD*, Δ*nfo*, Δ*mutM*, Δ*ung*, Δ*recE*, Δ*recT*) into AL-*ldrD* Δ*recA* and its empty expression control. Persistence assays conducted on stationary-phase populations of all mutants revealed biphasic killing, and the persister levels observed were not significantly different from one another or from the strains devoid of just *recA* (Fig. 6A; Fig. S6A to C). After 5 h of OFL treatment, all cultures were subjected to recovery assays by plating on LB-agar plates with aTc for 1, 2, 3, or 4 h. All double deletion mutants exhibited toxin-assisted rescue that was comparable to that of *ΔrecA* mutants (Fig. S6D and E), except for AL-*ldrD ΔrecA ΔuvrD*, which exhibited similar levels of survival with or without toxin induction (Fig. 6B; Fig. S6F). Growth assays confirmed that the toxin expression cassettes were functioning as expected in all of the double deletion mutants (Fig. S6G and H). Similarly, we observed comparable levels of OFL persisters between AL-*mazE*-*mazF* and AL-*mazE*-empty strains devoid of *recA* only and ones devoid of both *recA* and *uvrD* (Fig. 6C), but toxin-assisted recovery of AL-*mazE*-*mazF* Δ*recA* was absent when *uvrD* was also deleted (Fig. 6D). Interestingly, when we assayed strains with *recA* but devoid of *uvrD* (AL-*mazE*-*mazF* Δ*uvrD*, AL*-ldrD* Δ*uvrD*, and their respective controls), Δ*uvrD* strains exhibited 10-fold less killing than Δ*recA* Δ*uvrD* strains under OFL, but toxin-assisted rescue was still present in the Δ*uvrD* strains (Fig. 6E to H). To confirm that these results were directly attributable to the genetic deletions of *recA* and *uvrD*, we conducted complementation assays. Plasmid-borne expression of *uvrD* from its native promoter restored toxin-assisted rescue to Δ*recA* Δ*uvrD* strains, whereas the empty vector did not (Fig. S7A to D). In addition, plasmid-borne expression of *recA* from its native promoter in Δ*recA* Δ*uvrD* strains restored survival after 5 h of OFL treatment to levels observed for the Δ*uvrD* mutant alone and toxin-assisted rescue was also restored, whereas the empty vector could do neither (Fig. S7A to D). These data suggested that in a Δ*recA* background, *uvrD* was required to observe toxin-assisted rescue, whereas in a *recA*-proficient background, *uvrD* was not required, which indicated that both *recA*-dependent and -independent processes could facilitate survival post-FQ treatment.

10.1128/mBio.01983-21.6 FIG S6 Δ*uvrD* Δ*recA* strains do not undergo toxin-assisted recovery. Stationary-phase cultures of toxin-inducible (AL-*ldrD*) and control (AL-empty) strains harboring Δ*ung* Δ*recA*, Δ*nfo* Δ*recA*, Δ*mutM* Δ*recA*, Δ*recE* Δ*recA*, Δ*recT* Δ*recA*, and Δ*uvrD* Δ*recA* double DNA repair mutants were treated with 5 μg/ml OFL. All strains portrayed in this figure were not cured of their kanamycin resistance markers. (A to C) During the persister assay, all double DNA repair mutants exhibited similar killing during OFL treatment. (D and E) At the end of the persister assay, samples were washed three times with PBS and plated on LB agar plates with or without 3 ng/ml aTc. At 0, 1, 2, 3, and 4 h, samples were transferred to LB agar and incubated for 24 h at 37°C. Δ*ung* Δ*recA*, Δ*nfo* Δ*recA*, Δ*mutM* Δ*recA*, Δ*recE* Δ*recA*, Δ*recT* Δ*recA* mutants that were expressing LdrD showed an approximately 100-fold increase in survival during toxin-assisted recovery. These values were similar to ones observed in Δ*recA* mutants. (F) However, there was no observed recovery in the Δ*uvrD* Δ*recA* strains with toxin induction. (G and H) Toxin-inducible (G) and control (H) strains harboring either *ung*, *nfo*, *mutM*, *recE*, *recT*, or *uvrD* deletions along with *recA* deletions were grown in Gutnick glucose medium with 3 ng/ml aTc, and OD_600_ measurements were performed. All toxin-expressing mutants were growth inhibited with the addition of aTc, whereas their associated controls continued to grow. Download FIG S6, TIF file, 0.3 MB. Copyright © 2021 Lemma and Brynildsen. 2021 Lemma and Brynildsen. https://creativecommons.org/licenses/by/4.0/ This content is distributed under the terms of the Creative Commons Attribution 4.0 International license.

10.1128/mBio.01983-21.7 FIG S7 Complementation of UvrD or RecA restores toxin-assisted recovery in Δ*uvrD* Δ*recA* strains. (A to C) Δ*uvrD* Δ*recA* cells with pUA66 plasmid containing P_uvrD_-*uvrD* or P_recA_-*recA* and Δ*uvrD* Δ*recA* cells containing an empty plasmid control were treated with 5 μg/ml OFL for 5 h. All samples exhibited biphasic killing. (D to F) For recovery assays, at 5 h post-OFL treatment, cells were washed with PBS to reduce the concentration of OFL to below the MIC and were plated on filters placed on LB agar plates that contained aTc (3 ng/ml for AL-*ldrD* and AL-empty; 100 ng/ml for AL-*mazE*-*mazF* and AL-*mazE*-empty) or 25 μg/ml CM for MG1655. Cells were incubated on those plates for 1, 2, 3, or 4 h before being transferred to LB agar (AL-*ldrD*, AL-empty, and MG1655) and LB agar with 100 mM arabinose (AL-*mazE*-*mazF* and AL-*mazE*-empty). Complementation of *uvrD* or *recA* in Δ*uvrD* Δ*recA* strains restored toxin- and CM-assisted recovery. Empty vector controls showed no increase in survival with toxin induction or CM. An asterisk indicates statistical significance (*P* < 0.05) with respect to the same sample at *t* = 0 h. Download FIG S7, TIF file, 0.4 MB. Copyright © 2021 Lemma and Brynildsen. 2021 Lemma and Brynildsen. https://creativecommons.org/licenses/by/4.0/ This content is distributed under the terms of the Creative Commons Attribution 4.0 International license.

### Deletion of SOS toxins does not alter persistence of stationary-phase populations.

**FIG 7:**
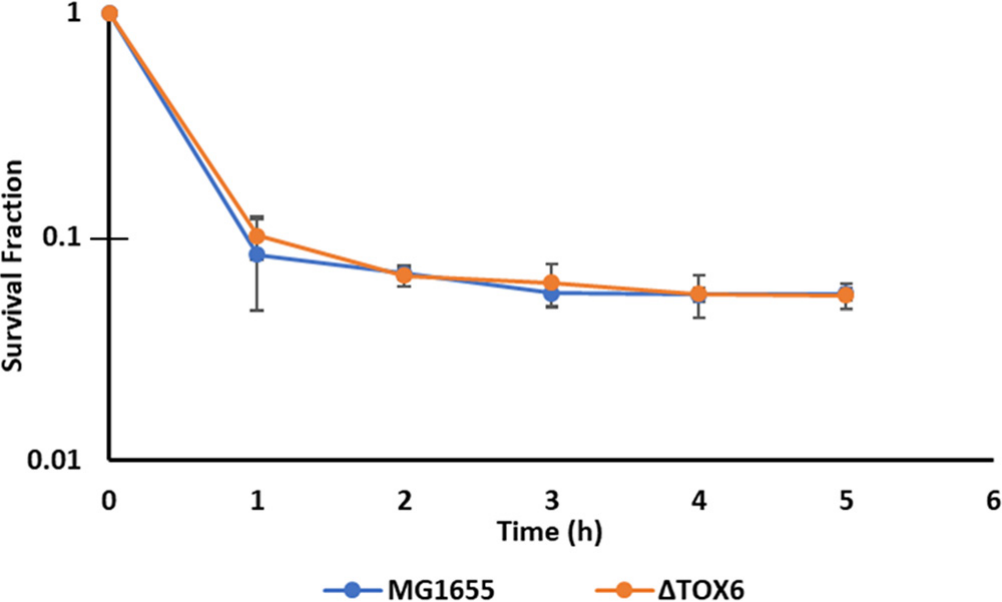
Deletion of six SOS toxins does not alter persistence of stationary-phase populations. Stationary-phase E. coli strains were treated with 5 μg/ml OFL for 5 h. At the indicated time points, samples were obtained, washed in PBS three times, and plated on LB agar. Wild-type MG1655 cells and a strain devoid of six known SOS-induced toxins (ΔTOX6) had comparable persistence levels.

Given the results with synthetic expression of toxins following FQ treatment (Fig. 3 to 6), we sought to assess whether a similar phenomenon occurred in wild-type strains. We reasoned that SOS-induced toxins might function in this manner for stationary-phase populations, since they would be induced largely after FQ treatment had ended and when populations were exposed to nutrients (26). To test this hypothesis, we deleted *hokE*, *symE*, *yafO*, *yafQ*, *tisB*, and *dinQ* (29) from the E. coli genome to generate the ΔTOX6 strain. Whole-genome sequencing confirmed that the strain was free of unwanted mutations (see Materials and Methods). Persistence assays of stationary-phase cultures of the ΔTOX6 strain revealed survival comparable to that of the wild type (Fig. 7). These results suggested that *hokE*, *symE*, *yafO*, *yafQ*, *tisB*, and *dinQ* do not influence FQ persistence in stationary-phase cultures.

### Inhibition of translation following OFL treatment rescues E. coli.

**FIG 8:**
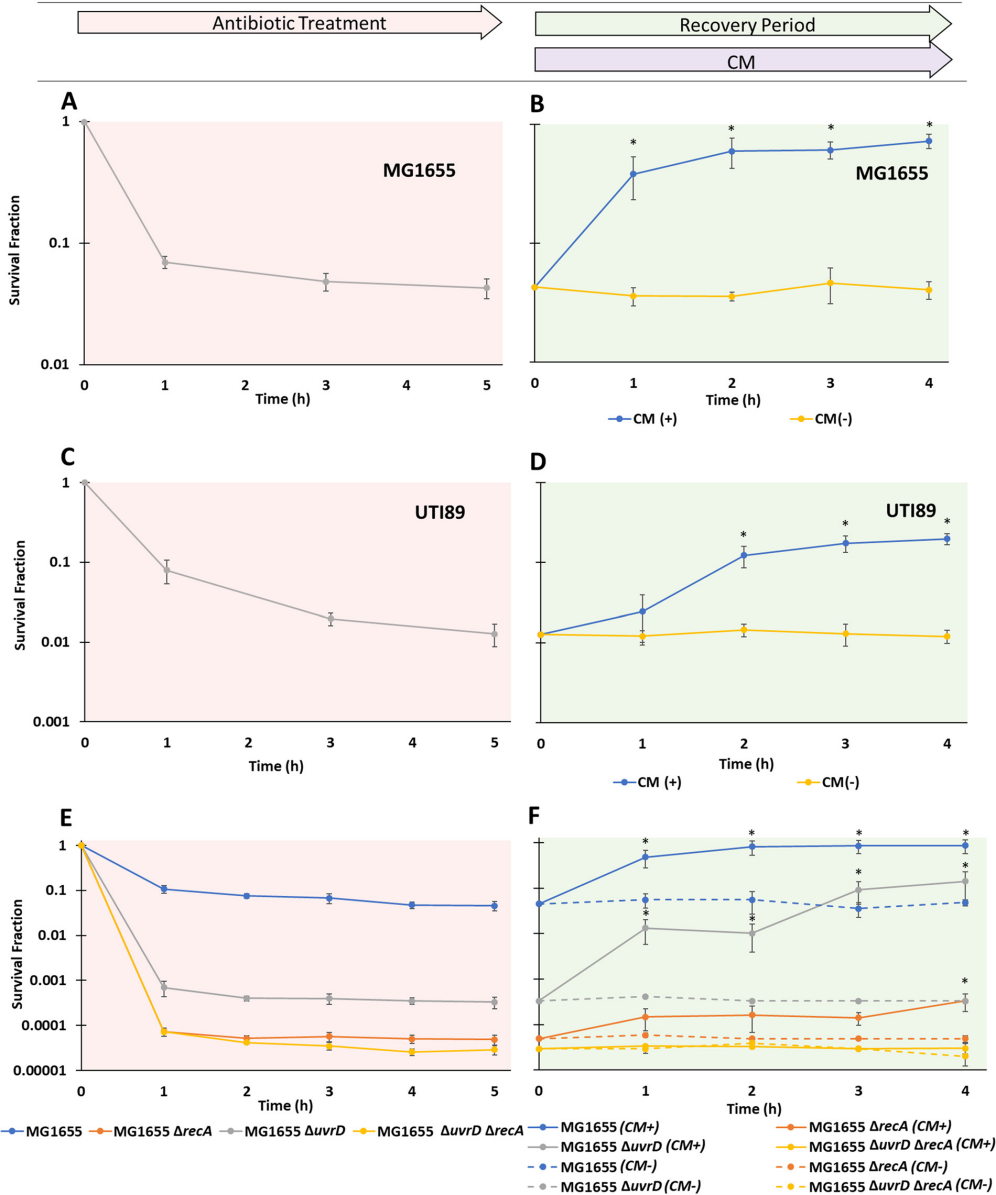
Inhibition of translation following OFL treatment rescues E. coli. (A) Stationary-phase E. coli MG1655 was treated with 5 μg/ml OFL, the numbers of CFU/ml were monitored, and survival fractions were calculated. A biphasic kill curve was observed. (B) At 5 h post-OFL treatment, cells were deposited on filters placed on top of LB agar plates with and without 25 μg/ml CM (MG1655). Samples were incubated on CM-containing or CM-free LB agar plates at 37°C for 1, 2, 3, or 4 h before being transferred to LB agar plates without CM. For MG1655, CM-assisted recovery resulted in significant increases in survival that reached ∼9-fold. (C) Stationary-phase uropathogenic E. coli UTI89 samples treated with 5 μg/ml OFL for 5 h exhibited a biphasic kill curve. (D) After 5 h of OFL treatment, cells were washed and plated on filters placed on top of LB agar plates with or without 50 μg/ml CM. After incubation for 1, 2, 3, or 4 h at 37°C, the samples were transferred to LB agar plates without CM. CM-assisted recovery for UTI89 resulted in significant increases in survival that reached ∼15-fold at 4 h. (E) Stationary-phase MG1655 strains devoid of *recA*, *uvrD*, and both *recA* and *uvrD*, as well as the wild-type control, were treated with 5 μg/ml OFL for 5 h, and their CFU/ml were enumerated. All samples exhibited biphasic killing. (F) Subsequent recovery assays were conducted on MG1655 and its Δ*recA*, Δ*uvrD*, and Δ*uvrD* Δ*recA* mutants where 5-h OFL-treated samples were plated on filters placed on top of LB agar plates with or without 25 μg/ml CM for 1, 2, 3, or 4 h before being transferred to LB agar plates without CM. With CM-assisted recovery, for Δ*recA* mutants, we observed a statistically significant increase in survival that was ∼7-fold at *t* = 4 h, whereas for Δ*uvrD* mutants, statistically significant increases in survival that reached ∼400-fold were observed. A strain devoid of both *recA* and *uvrD* did not exhibit any increase in survival. Data points indicate the mean values of the results of at least three biological replicates with error bars indicating standard errors. An asterisk indicates statistical significance (*P* < 0.05) with respect to the same sample at *t* = 0 h.

As a second approach to test the applicability of toxin-assisted rescue to the wild type, we reasoned that chemical inhibition of the same macromolecular processes targeted by toxins might rescue wild-type cells from FQ-mediated cell death. Inspired by MazF, which impairs translation, we treated stationary-phase cultures with OFL for 5 h, which produced the prototypical biphasic killing kinetics (Fig. 8A), and then washed and plated cells on filters on top of LB agar plates with or without 25 μg/ml of CM. Interestingly, CM increased survival of OFL-treated, stationary-phase populations by ∼9 fold to approximately ∼80%, which was much like LdrD and MazF expression during the recovery period (Fig. 8B). To ascertain whether a similar phenomenon can be observed for pathogenic bacteria, we performed analogous experiments with uropathogenic E. coli (UPEC) strain UTI89. Biphasic killing was observed during persistence assays with UTI89 (Fig. 8C), and recovery assays revealed that CM increased survival by ∼15 fold, leading to approximately ∼20% of the UTI89 population surviving OFL treatment (Fig. 8D). These data suggest that the FQ persistence of stationary-phase populations of pathogenic and nonpathogenic E. coli can be significantly increased if translation is inhibited temporarily following treatment. Further, similar to the toxin-assisted recovery, *uvrD* and *recA* were integral for the ability of CM to rescue FQ-treated E. coli (Fig. 8E and F). We observed that in a Δ*recA* background, *uvrD* was required to observe rescue, whereas with a functional *recA*, *uvrD* was not required to observe rescue (Fig. 8F). Complementation experiments in which *uvrD* or *recA* was expressed from their native promoters confirmed the conditional dependency of each in this phenomenon (Fig. S7E and F). These data suggested that toxin-assisted recovery and CM-assisted recovery share the same pathway.

### Inhibition of transcription following OFL treatment rescues E. coli.

**FIG 9:**
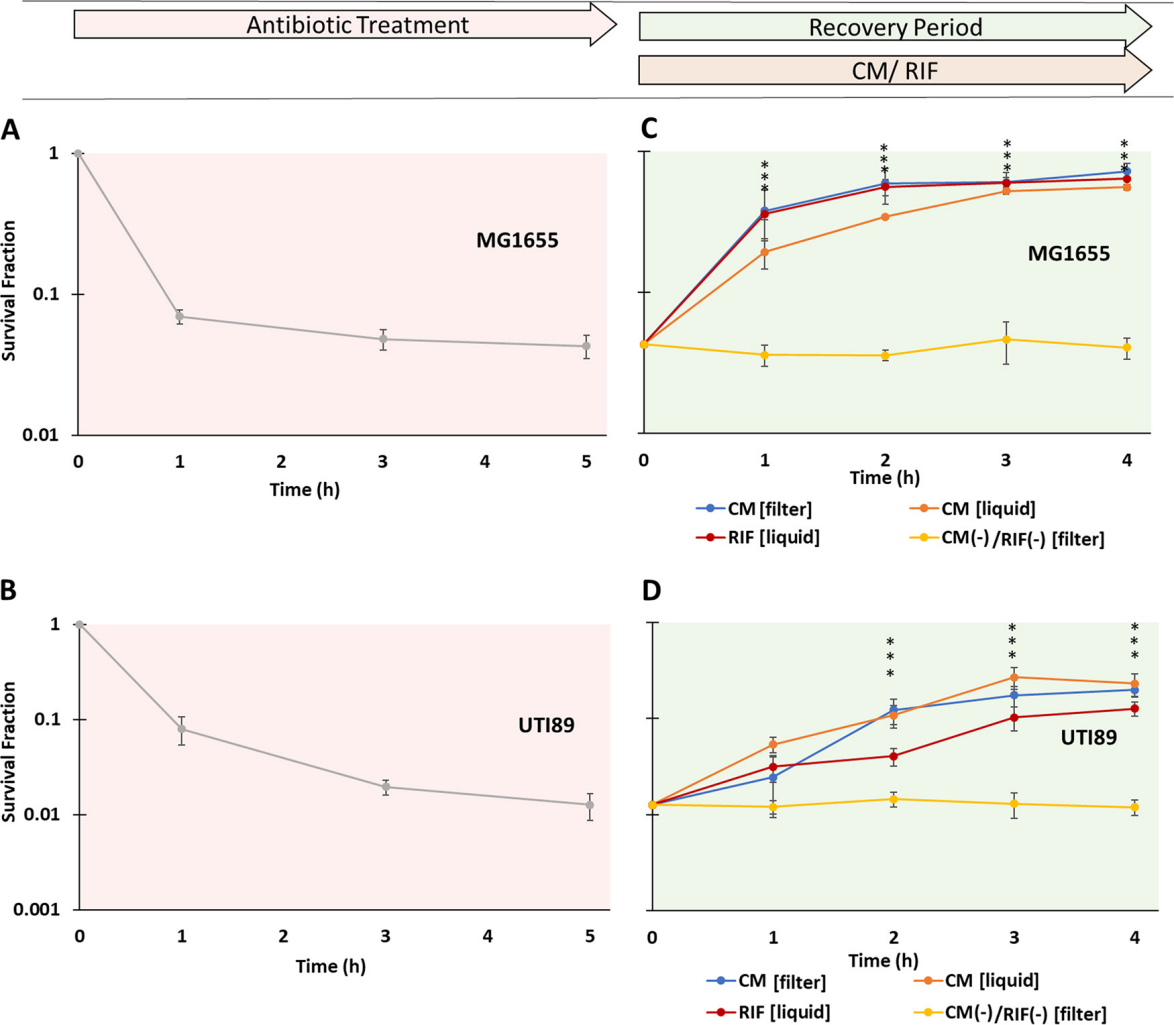
Transcriptional inhibition post-OFL treatment increases survival. (A and B) Stationary-phase E. coli MG1655 and uropathogenic E. coli UTI89 were treated with 5 μg/ml OFL for 5 h, and their survival was monitored. Biphasic kill curves were observed for both strains. (C and D) For recovery assays, 5-h OFL-treated cultures were washed with PBS and resuspended in LB with 100 μg/ml RIF. As a control for the liquid-based recovery protocol, CM-assisted recovery was conducted in parallel on filters as well as in liquid (25 μg/ml CM for MG1655 and 50 μg/ml CM for UTI89) (see Materials and Methods). At 1, 2, 3, and 4 h, the samples were washed (for liquid cultures), transferred to LB agar plates, and incubated for 24 h, after which CFU were enumerated. (C) An ∼15-fold increase in survival was observed for RIF-assisted recovery of MG1655 at *t* = 4 h. (D) An ∼10-fold increase in survival was observed for RIF-assisted recovery of UTI89. Importantly, the ability of CM to facilitate recovery in the filter-based protocol was equivalent to that in the liquid-based protocol for both strains. Experiments with RIF were performed using the liquid-based protocol, because RIF absorbed noticeably into the membranes and thus was carried over to plate 2 (Fig. 2) in considerable amounts. Results for non-OFL-treated controls are provided in Fig. S8 in the supplemental material. An asterisk indicates statistical significance (*P* < 0.05) with respect to the same sample at *t* = 0 h.

After we observed that post-FQ translational inhibition resulted in significant increases in survival, we sought to determine whether transcriptional inhibition resulted in the same outcome. However, the membrane filters used to conduct the recovery assays exhibited high affinity to RIF, which produced growth issues when the filters were transferred from plate 1 to plate 2 (Fig. 2A). To circumvent this issue, we tested whether using liquid medium in place of plate 1 gave equivalent results for CM. As depicted in Fig. 9, we observed that the results for CM between the two methods was indistinguishable. Using liquid-based exposure to RIF after FQ treatment, followed by washing and plating on LB agar, significant increases in survival for E. coli MG1655 and UTI89 that were comparable to those obtained with CM were observed (Fig. 9). Recovery assays performed on controls not treated with OFL showed that RIF and CM treatments prevented cell division from contributing to culturability measurements during recovery assays in liquid medium (Fig. S8). These results demonstrated that inhibition of transcription, like translation, following the conclusion of FQ treatment can significantly increase the survival of pathogenic and nonpathogenic E. coli.

10.1128/mBio.01983-21.8 FIG S8 RIF and CM liquid recovery assays show increases in survival only on OFL-treated samples. Control experiments were conducted on 20-h stationary-phase cultures treated with autoclaved water instead of OFL. After 5 h of water treatment, these samples were diluted 40-fold for MG1655 and 100-fold for UTI89 so they would be at the same culturable density as OFL-treated samples before being incubated in LB containing 100 μg/ml RIF or CM (25 μg/ml for MG1655 and 50 μg/ml for UTI89). At 1, 2, 3, or 4 h, the samples were washed with PBS three times and plated on LB agar plates. RIF and CM treatment of stationary-phase cultures that were treated with water in place of OFL did not exhibit any significant change in survival. Download FIG S8, TIF file, 0.2 MB. Copyright © 2021 Lemma and Brynildsen. 2021 Lemma and Brynildsen. https://creativecommons.org/licenses/by/4.0/ This content is distributed under the terms of the Creative Commons Attribution 4.0 International license.

## DISCUSSION

A toxin was first associated with the persistence phenotype when a mutant that conferred high levels of persistence, the *hipA7* mutant, was acquired after successive rounds of AMP treatment (12). In that strain, the HipA toxin harbored G22S and D291A mutations, which reduced the stability of its interaction with its antitoxin, HipB (40). The mechanism of HipA toxicity was found to be through phosphorylation of GltX, a glutamate-tRNA ligase, which led to translational inhibition (14). Importantly, the *hipA7* allele was found in 23 of 477 patient isolates, of which some were associated with urinary tract infections (UTI), and deletion of *hipA7* from a clinical UTI isolate showed a significant decrease in CIP persister levels (41). These data provided strong evidence that toxins can be involved in persistence and that their impacts on the phenotype can be observed in clinical isolates.

For any discussion on toxins and persistence, it requires mention that some previous works linking TA systems and persister formation have been retracted (42, 43) and that the contribution of TA modules to persistence has become more tenuous (44–46). Despite these developments, toxin-expressing cells have provided valuable model systems for understanding how antibiotic-tolerant cells, such as persisters, survive treatment (2, 27, 35). For example, a study that used a *hipA7* mutant revealed that persisters cells that survived AMP treatment exhibited slowed growth before being exposed to antibiotics (2). Using bacteria whose growth had been arrested with MazF, we observed that the relative timing of DNA damage responses and growth resumption following FQ treatment were important to survival and found that the same was true for wild-type cells (27). Further, TisB and HokB overexpression that resulted in pore formation, membrane depolarization, and ATP leakage helped elucidate the potential role of ATP depletion and cellular energy level in the formation of wild-type persisters (35, 36). These studies demonstrated that toxin-arrested cells can provide insight into antibiotic-tolerant mechanisms; however, an outstanding question has been to what extent toxin activity after antibiotic treatment can contribute to survival.

Here we sought to assess whether toxin expression following antibiotic treatment can alter persistence. The antibiotic class selected was FQs, and this was inspired by previous work establishing the importance of the postantibiotic recovery period to FQ persister survival in nongrowing cultures (26, 27) and knowledge that FQs induce the SOS response, which includes six TA modules, in stationary-phase populations largely during the recovery period (26). The toxins selected were MazF, because it was a toxin from a type II system with which we had previously worked (30), and LdrD, because it was a toxin from a type I system, which is conserved in enteric bacteria (e.g., E. coli, Salmonella enterica serovar Typhimurium, and Citrobacter freundii) yet remains minimally studied (28). Importantly, LdrD model persisters and MazF model persisters required similar DNA repair enzymes to survive FQ treatment when compared to the wild type (see Fig. S2D and E in the supplemental material and reference 30), which suggested that analysis of these model systems may shed light on persistence mechanisms of the wild type.

With both the LdrD and MazF model systems, we demonstrated that toxin induction post-FQ treatment of stationary-phase cultures resulted in significant increases in survival that approached levels obtained with expression before and during treatment (Fig. 4). These data suggested that the tolerant-inducing action of toxins is not confined just to before and/or during treatment but that their activities after treatment can be equally important for FQ treatment of nongrowing bacteria. Based on these observations, we hypothesized that endogenous SOS toxins might function in this manner (11, 47, 48). The E. coli SOS response includes toxins from both type I (*tisB/istR-1*, *symE/symR*, *dinQ/argB*, *hokE/sokE*) and type II (*yafNO*, *dinJ/yafQ*) systems (11, 47–50), and SOS induction of TisB has been implicated in persistence to CIP in exponential-phase cultures (7), although its involvement in FQ persistence has not been observed for stationary-phase populations (7, 26). To test our SOS toxin hypothesis, we deleted all six SOS toxin genes and measured persistence to FQs in stationary-phase cultures. The ΔTOX6 mutant had persister levels comparable to that of the wild type (Fig. 7), which suggested that those toxins were not involved in defining the FQ persistence level of the stationary-phase E. coli populations used here. In recognition that the macromolecular processes targeted by toxins can be inhibited by chemical agents, we revisited our hypothesis and asked whether chemical inhibition of transcription or translation following FQ treatment functions similarly to toxin induction to facilitate survival. Using CM and RIF to inhibit translation and transcription only after FQ treatment of nongrowing populations, we observed significant increases in survival in E. coli MG1655 as well as UPEC strain UTI89. These data suggested that transcriptional or translational inhibition following FQ treatment, independent of the cause (toxin or chemical), can increase the survival of nongrowing bacterial populations, which remain the most difficult to treat with antibiotics. It is important to note that posttreatment toxin induction was not able to rescue FQ-treated, exponential-phase cultures (Fig. S4A and B), just as post-FQ starvation could not rescue cells treated with FQ during exponential growth in a previous study (27). We postulate that this occurs because DNA damage and repair happen simultaneously with growth-related processes in growing cultures, whereas with populations that are growth inhibited during FQ exposure, starvation, toxin induction, and chemical inhibition of transcription or translation delays growth-related processes, which thereby facilitates DNA repair and survival. An outstanding question that these data raise is the following: for toxin-arrested cells in what would be growth-promoting environments, to what extent does post-FQ toxin activity contribute to survival? Data from Fig. S4A and B depict that for growth-promoting environments, toxin activity post-FQ treatment is not sufficient to enhance survival, but those data did not answer the question of whether it was necessary. Experiments with rapid toxin inactivation post-FQ treatment will be necessary to answer that question in future studies.

RecA and UvrD both resulted in significant decreases in persister levels when deleted independently, with Δ*recA* strains exhibiting an ∼10- to 20-fold greater decrease in persisters than Δ*uvrD* strains under FQ treatment, which is consistent with previous results (27, 39). During recovery assays, significant increases in survival were observed for Δ*recA* as well as Δ*uvrD* strains (Fig. 5C and D and 6F and H); however, those recovery-based enhancements in survival were absent from Δ*recA* Δ*uvrD* strains. RecA is involved in homologous recombination and is integral to the double-stranded DNA break repair pathway (51). It is also part of the SOS response induced after DNA damage, as it facilitates the autocatalytic cleavage of the LexA repressor and is among those genes induced when LexA repression is lifted (52). UvrD has been found to be involved in a number of RecA-dependent and RecA-independent repair pathways (39, 53–55). UvrD takes part in the MutSHL mismatch repair pathway, the UvrABC nucleotide repair pathway, and the UvrAB replication backup system for DNA polymerase I, as well as the UvrABC pathway for removal of stalled RNA polymerase (39, 53–55). Although the basis of the RecA and UvrD epistasis observed here was not elucidated, we anticipate that further experiments with additional DNA repair and SOS response mutants will unravel their interaction that significantly increases the persistence of FQ-treated populations. One possibility involves redundant repair pathways whose prominence depends on the presence or absence of the other mechanism.

Bacterial persisters are implicated in the recalcitrance of chronic infections (56, 57) and can serve as reservoirs for the emergence of resistant mutants (38, 58, 59). Pharmacokinetic modeling has predicted that persisters can lead to reduced efficacy of antibiotic treatments, prolonged duration of infection, and ultimately treatment failure (58). As the clinical relevance of persisters continues to accumulate (60–62), the need to understand what enables their survival becomes more evident. Utilizing mechanistically different model persister systems, we were able to observe that post-FQ treatment toxin induction can result in an increase in bacterial survival for cultures that were growth inhibited at the time of antibiotic exposure. Translating this finding to wild-type cells revealed that impeding the ability of cells to translate or transcribe after the removal of an FQ can lead to near complete survival. This highlights how important it is to consider the environment surrounding bacteria after treatment in order to get a full sense of what defines bacterial life or death after antibiotic treatment.

## MATERIALS AND METHODS

### Bacterial strains and plasmids.

All strains were derived from E. coli MG1655, except for UTI89, and details are provided in Table S1 in the supplemental material. Genetic mutations were incorporated by P1 phage transduction or the method of Datsenko and Wanner (63, 64). To facilitate the integration of an inducible expression cassette for *ldrD*, the plasmid pTOX66 bearing a tetracycline-inducible promoter (P_LtetO1_), a tetracycline repressor (*tetR*), a gentamicin resistance gene (*gentR*), and homologous regions for integration between *ybbD* and *ylbG* was used (30). The *ldrD* open reading frame (ORF) was amplified from E. coli MG1655 genomic DNA, digested with KpnI and EcoRI, and cloned into pTOX66. The expression cassette and homologous regions were amplified using the plasmid as a template and integrated into E. coli MOΔ*ldrD*/*rdlD* using the Datsenko-Wanner method (64). To construct the control strain (devoid of *ldrD*), the unmodified pTOX66 plasmid was used as a template, and the amplified sequence was integrated into E. coli MOΔ*ldrD*/*rdlD* using the Datsenko-Wanner method (64). The AL-*mazE*-*mazF* strain was constructed in a previous study (30), whereas AL-*mazE*-empty was constructed by using the unmodified pTOX66 plasmid as a template and integrating the amplified sequence into the MO::*mazE* strain (Fig. S1). Genetic mutations and plasmids were confirmed by PCR and/or sequencing (Genewiz, South Plainfield, NJ) with the primers provided in Table S2. When necessary, kanamycin resistance markers were removed using FLP recombinase expressed from pCP20. In the case of ΔTOX6, the strain was generated by P1 phage transduction of mutations from the Keio collection (65) (*hokE*, *symE*, *tisB*, *yafQ*) as well as by the Datsenko-Wanner method (64) when the genes to be deleted were <100 kb away from previous mutations (*yafO*, *dinQ*). At each step, kanamycin resistance was removed by FLP recombinase and PCR was used to assess the presence or absence of accumulating mutations. Upon completion of ΔTOX6 strain generation, whole-genome sequencing was performed (see below). For complementation of *uvrD* and *recA*, pUA66 plasmids containing either P_recA_-*recA* (38) or P_uvrD_-*uvrD* were used. For the construction of pUA66 P_uvrD-_*uvrD*, the P_uvrD_-*uvrD* region was amplified from MG1655 genomic DNA using primers harboring restriction enzyme sites listed in Table S2. The amplified insert and pUA66 vector were then digested with XhoI and SbfI, ligated, and transformed into XL1-Blue cells. Table S1 includes a list of strains and plasmids, and Table S2 includes a list of oligonucleotides used in this study.

10.1128/mBio.01983-21.9 TABLE S1 Bacterial strains and plasmids. Download Table S1, DOCX file, 0.03 MB. Copyright © 2021 Lemma and Brynildsen. 2021 Lemma and Brynildsen. https://creativecommons.org/licenses/by/4.0/ This content is distributed under the terms of the Creative Commons Attribution 4.0 International license.

10.1128/mBio.01983-21.10 TABLE S2 DNA oligonucleotides. Download Table S2, DOCX file, 0.02 MB. Copyright © 2021 Lemma and Brynildsen. 2021 Lemma and Brynildsen. https://creativecommons.org/licenses/by/4.0/ This content is distributed under the terms of the Creative Commons Attribution 4.0 International license.

### Chemicals and media.

All media were made with distilled water purified using a Millipore Milli-Q lab water system (Burlington, MA) to a resistivity of 18.2 MΩ·cm (MilliQ water). All chemical components were obtained from Fisher Scientific or Sigma-Aldrich, unless specified otherwise. LB medium consisted of 10 g/liter tryptone, 5 g/liter yeast extract, and 10 g/liter NaCl dissolved in MilliQ water, which was then autoclaved. Minimal Gutnick glucose medium contained 10 mM glucose, 10 mM NH_4_Cl, and 1× Gutnick salts in autoclaved water, which was then sterilized using 0.22-μm filters (Merck Millipore Ltd., Burlington, MA). A 10× Gutnick salts solution contained 47 g/liter KH_2_PO_4_, 135 g/liter K_2_HPO_4_, 10 g/liter K_2_SO_4_, and 1 g/liter MgSO_4_·7H_2_O in MilliQ water, which was autoclaved. LB agar plates were made with 25 g/liter premixed LB Miller broth (Fisher) and 15 g/liter agar dissolved in MilliQ water, which was autoclaved. For mutant selection and plasmid maintenance, 100 μg/ml AMP, 50 μg/ml KAN, or 15 μg/ml gentamicin (GENT) was used in medium and plates. For *ldrD* expression, 3 ng/ml of aTc was used, whereas for *mazF* expression, 100 ng/ml of aTc was used in medium or plates. For *mazE* expression, 100 mM arabinose was used (30). For translational inhibition, 25 μg/ml CM was used unless otherwise indicated. For the cell depolarization assay, 10 μg/ml of DiBAC_4_(3) [bis-(1,3-dibarbituric acid)-trimethine oxanol] was used, and cells treated with 100 μM carbonyl cyanide 3-chlorophenylhydrazone (CCCP) or 30% ethanol were used as controls. Stock solutions for all chemicals except aTc, CM, CCCP, and OFL were prepared in autoclaved MilliQ water. aTc and CM were dissolved in ethanol, CCCP was dissolved in dimethyl sulfoxide (DMSO), and 1 M NaOH was added to OFL stock solution in autoclaved MilliQ water until it was fully dissolved. All stock solutions were prepared in autoclaved MilliQ water and were sterile filtered before use. Sterile-filtered 1× phosphate-buffered saline (PBS) solution prepared from a 10× stock was used for all wash steps.

### Cell density measurement (OD_600_).

All OD_600_ measurements were performed on a Synergy H1 hybrid multimode microplate reader with flat-bottom, clear, 96-well plates and 300 μl in each well. Samples with an OD_600_ above 0.4 were diluted in cell-free medium such that their measured OD_600_ was well within the linear range of the spectrophotometer (0.01 to 0.4).

### Culture inoculations and conditions.

Cultures were first inoculated from −80°C, 50% glycerol stocks into 2 ml of LB medium in test tubes and incubated for 4 h at 37°C with shaking (250 rpm). After 4 h, those pregrowth cultures were diluted 100-fold in Gutnick medium with 10 mM glucose as the sole carbon source and incubated at 37°C with shaking (250 rpm) overnight for 16 or 20 h. The 16-h overnight cultures were used in the characterization of the LdrD model persister system (Fig. 1; Fig. S2), where they were diluted to an OD_600_ of 0.01 in 25 ml of Gutnick medium with 10 mM glucose in 250-ml baffled flasks and grown to an OD_600_ of 0.1. At an OD_600_ of 0.1, those exponential-phase cultures were diluted 5-fold to an OD_600_ of 0.02 into 25 ml of fresh Gutnick minimal medium for subsequent assays (Fig. 1; Fig. S2, S4A and B, and S6G and H). The 20-h overnight cultures were used as stationary-phase cultures in other assays (Fig. 2C and D, 3, and 9; Fig. S3, S4C and D, S5, S6A to C, S7, and S8).

### Growth and culturability of LdrD model system.

Exponential-phase cultures diluted to an OD_600_ of 0.02 were treated with 0, 1, 2, or 3 ng/ml aTc and incubated at 37°C with shaking (250 rpm), and the OD_600_ was measured hourly. For culturability measurements, at *t* = 0, before the addition of aTc, and at 1, 2, 3, 4, and 5 h, after the addition of the inducer, 500-μl samples were removed. Those samples were washed three times with PBS by centrifuging the cells at 15,000 rpm for 3 min, removing 450 μl of the supernatant, and then resuspending the cell pellet in 450 μl of PBS. After three washes, the samples were then centrifuged again, followed by removal of 400 μl of supernatant and resuspension of the cell pellet in the remaining 100 μl of PBS. That 5-fold-concentrated sample was then serially diluted in PBS, plated on LB agar, and incubated at 37°C for 16 h (Fig. 1A and B).

### Plate-based growth assay for model persistence systems.

To assess the effects of toxin induction in plate 1 (Fig. 2A and B), stationary-phase cultures of AL-*ldrD*, AL-empty, AL-*mazE*-*mazF*, and AL-*mazE*-empty were plated on LB agar plates with or without aTc (3 ng/ml aTc for AL-*ldrD* and AL-empty and 100 ng/ml aTc for AL-*mazE*-*mazF* and AL-*mazE*-empty) and incubated at 37°C for 16 h (Fig. 2C and D).

### DiBAC_4_(3) assay.

Exponential-phase cultures diluted to an OD_600_ of 0.02 were used to conduct cell depolarization measurements. For samples treated with CM, 50 μg/ml was added at *t* = 2 h. At *t* = 0 (before aTc or CM addition) and at *t* =3 h, samples were collected and cell density was adjusted to ∼5 × 10^6^ CFU/ml in 500 μl of Gutnick minimal medium with 10 mM glucose. For a control, AL-empty and AL-*ldrD* cells were treated with 30% ethanol or 100 μM CCCP for 15 min before DiBAC_4_(3) was introduced. All samples were then incubated in 10 μg/ml of DiBAC_4_(3) in the dark at 25°C for 15 to 20 min. Fluorescence of the samples was measured by flow cytometry using a LSRII flow cytometer. Dye uptake was measured fluorometrically with excitation at 488 nm and emission using a 525/50-nm bandpass filter. The resulting data were analyzed using FACSDiva software (Fig. S2A).

### ATP measurements.

Extracellular and total ATP content were measured using the BacTiter-Glo microbial cell viability assay (Promega) by following the manufacturer’s instructions. Exponential-phase cultures diluted to an OD_600_ of 0.02 were used. At *t* = 0 (before aTc addition) and at *t* =1, 2, and 3 h, samples were collected and spun down for 1 min at 15,000 rpm. For extracellular ATP measurement, the supernatant from the samples was sterile filtered and the medium flowthrough was used for analysis. For total ATP measurement, the pelleted cells were resuspended in the same medium for analysis. One hundred microliters of the sample or its flowthrough medium was mixed with 100 μl of BacTiter-Glo reagent. A standard curve with known ATP concentrations (0, 0.125, 0.25, 0.5, and 1 μM) was prepared using an ATP stock solution diluted in the sterile-filtered medium. Intracellular ATP was calculated to be the difference between total sample ATP and extracellular ATP measurements (Fig. S2B and C).

### SOS reporter measurements.

AL-empty and AL-mazE-empty strains containing an SOS reporter plasmid (pUA66 with P_recA_-*gfp*) were treated with OFL for 5 h. Following treatment, cells were washed with PBS three times and diluted 250-fold into 25 ml of recovery medium (LB with 50 μg/ml KAN for plasmid retention). At *t* = 0 h (before inoculation) and at *t* = 1, 2, 3, 4, 5, 6, 7, and 8 h in recovery medium, OFL-treated and untreated samples were collected, washed in PBS, and resuspended in 500 μl of 4% paraformaldehyde (PFA) for fixation. After 15 min, cells were pelleted, the supernatant was removed, and the cell pellet was resuspended in 500 μl of PBS. Fluorescence of the samples (excitation, 488 nm; emission, 525 nm; with a 50-nm bandpass filter) was measured using an LSRII flow cytometer. Forward scatter (FSC) and side scatter (SSC) parameters were used to identify single cells, and the resulting data were analyzed using FACSDiva software (Fig. 2E and F; Fig. S3).

### Persistence assays.

The following procedure was used to quantify persister levels. Five-hundred-microliter samples were removed for *t* = 0 h measurements before antibiotic was introduced. After antibiotic treatment (5 μg/ml OFL, 100 μg/ml AMP, 1 μg/ml CIP, or 5 μg/ml MOX), cultures were incubated at 37°C with shaking (250 rpm) and 500-μl samples were taken at 1, 3, and 5 h. Samples at all time points were washed three times by pelleting the cells (centrifugation at 15,000 rpm, 3 min), followed by removal of 450 μl of the supernatant and resuspension in 450 μl of sterile PBS. After the three washes in PBS, samples were concentrated by removing 400 μl of supernatant and resuspending the remaining cells in the 100 μl PBS, which produced a 5-fold concentration of samples. Ten microliters of the concentrated samples was used for serial dilutions in 90 μl of PBS. Ten microliters per dilution was spotted onto LB agar, and the plates were incubated at 37°C for 24 h, after which CFU were measured. Persister levels were those within the second, slower phase of the kill curve. Persistence assays were conducted using three different types of culture. The first, which established AL-*ldrD* as a system capable of generating model persisters, were cultures that were or were not treated with 3 ng/ml aTc at an initial OD_600_ of 0.02 in Gutnick medium with 10 mM glucose for 5 h (Fig. 1C and D). The second, which constitutes the majority of cultures used in persistence assays performed in this study, were stationary-phase cultures that had been grown for 20 h in Gutnick medium with 10 mM glucose. The third, used for experiments in which toxins were induced prior to or during antibiotic treatment (Fig. 4A and B), were stationary-phase cultures that were diluted 100-fold in spent medium and in which 3 ng/ml aTc (LdrD) or 100 ng/ml aTc (MazF) was introduced at *t* = 18 h (before) or *t* = 20 h (during) of growth. The dilution was done to approximate the cell density at which the persister model systems had been characterized to yield growth inhibition from toxin expression (Fig. 4A and B) (30). For the “before” samples, cells continued to be incubated at 37°C with shaking (250 rpm) until *t* = 20 h, at which point the persistence assay was initiated, whereas for the “during” samples, antibiotic was added at the time of the dilution.

### Recovery assay.

At the conclusion of persistence assays (after 5 h of FQ treatment), cells were washed three times with PBS, and with each wash, cells were centrifuged at 15,000 rpm for 3 min, 450 μl of the supernatant was removed, 450 μl of sterile PBS was introduced, and the cell pellet was resuspended. After these wash steps, samples were serially diluted in PBS, and three dilutions, 10 μl of each, were inoculated onto Supor 200 polyethersulfone membranes with 0.2-μm pores (Pall Corporation), placed on top of LB agar plates with or without 3 ng/ml aTc (AL-*ldrD*, AL-empty), 100 ng/ml aTc (AL-*mazE*-*mazF*, AL-*mazE*-empty), 25 μg/ml CM (MG1655), or 50 μg/ml CM (UTI89) and incubated at 37°C (Fig. 2A and B). As in previous work, membranes were used to immobilize cells so that any cell divisions during the recovery period did not alter the CFU/ml measurements. At *t* = 1, 2, 3, or 4 h, membranes were transferred to LB agar plates (with 100 mM arabinose for AL-*mazE*-*mazF* and AL-*mazE*-empty) and incubated at 37°C for 24 h. For strains harboring plasmids that required KAN for maintenance, 50 μg/ml KAN was provided. When RIF was used during the recovery assay on solid medium, visible absorption of RIF into the membranes was observed, and upon transfer to plate 2 (Fig. 2A and B), the RIF that carried over prevented growth. To address this issue, we adapted plate 1 of the recovery assay (Fig. 2A and B) to liquid medium, which enabled a wash step to be introduced before final inoculation onto plate 2. Importantly, we confirmed that the enhancement in survival that was achieved with CM using solid or liquid medium in the first step of the recovery assay was indistinguishable, which gave confidence that the liquid version of the assay could be used with RIF. Specifically, the adjustment in procedure was that instead of plating the cells on agar, the cells were resuspended in LB containing 100 μg/ml RIF or 25 μg/ml CM (or 50 μg/ml CM for UTI89) and incubated at 37°C and 250 rpm in test tubes for 1, 2, 3, and 4 h. At those time points, samples were collected and washed three times with PBS by centrifuging the cells at 15,000 rpm for 3 min, removing 450 μl of the supernatant, introducing 450 μl of sterile PBS, and resuspending the cell pellet. The samples were then serially diluted and plated on LB agar plates and incubated for 24 h. As a control, non-OFL-treated 20-h stationary-phase cultures were treated with autoclaved water in place of OFL for 5 h and the samples were diluted 40-fold for MG1655 and 100-fold for UTI89 to achieve the same culturable density as 5-h OFL-treated samples. The diluted samples were then incubated in LB containing 100 μg/ml RIF or CM (25 μg/ml for MG1655 and 50 μg/ml for UTI89). At 1, 2, 3, or 4 h, the samples were washed with PBS three times and plated on LB agar plates. RIF and CM treatment of stationary-phase cultures did not exhibit any significant change in survival, which showed that RIF and CM during liquid recovery assays prevented cell division from contributing to increases in CFU/ml measurements.

### Whole-genome sequencing.

Bacterial genomic DNA (gDNA) was extracted using Qiagen DNeasy blood and tissue kits (Qiagen, Inc., Germantown, MD). The gDNA samples were sequenced in a 300-nucleotide lane on a MiSeq (Illumina) sequencer. Raw sequencing reads were further analyzed using Galaxy (66–68). The reads were assembled using Unicycler (69) and mapped with BWA-MEM onto a reference E. coli chromosome (NCBI reference sequence NC_000913.2) (70, 71). The assembled sequence was visualized on IGV software and analyzed. The whole-genome sequence analysis confirmed the deletions of the six SOS toxin genes (*hokE*, *symE*, *tisB*, *yafQ*, *yafO*, and *dinQ*) in the ΔTOX6 strain. Both the parental MG1655 strain and the ΔTOX6 strain were found to harbor point mutations in *fdrA* (position 547694; A to G), *yzgL* (position 3563722; C to T), *ilvC* (position 3957957; T to C) and *mdtO* (position 4300545; A to T) when mapped onto the reference E. coli chromosome (NCBI NC_000913.2). There were no mutations, insertions, or deletions other than the six deleted TA systems observed in the ΔTOX6 strain in comparison to the parental MG1655 it was derived from.

### Statistical analysis.

For each experiment, three biological replicates were performed. An asterisk (*) indicates a pairwise comparison with a two-tailed *t* test and *P* values of  ≤0.05. The data points in the figures indicate the mean values of the results of at least three biological replicates, and the error bars indicate standard errors of the mean.

### Data availability.

Raw sequencing data of forward and reverse reads (two separate files in database) for the ΔTOX6 strain are available on Figshare data repository and can be accessed at https://doi.org/10.6084/m9.figshare.14904444.v1.
