# Supplementary figures and images for: Toxin Induction or Inhibition of Transcription or Translation Posttreatment Increases Persistence to Fluoroquinolones
Source: mBio. 2021 Aug 17;12(4):e01983-21. doi: 10.1128/mBio.01983-21 (PMC8406316; doi:10.1128/mBio.01983-21)

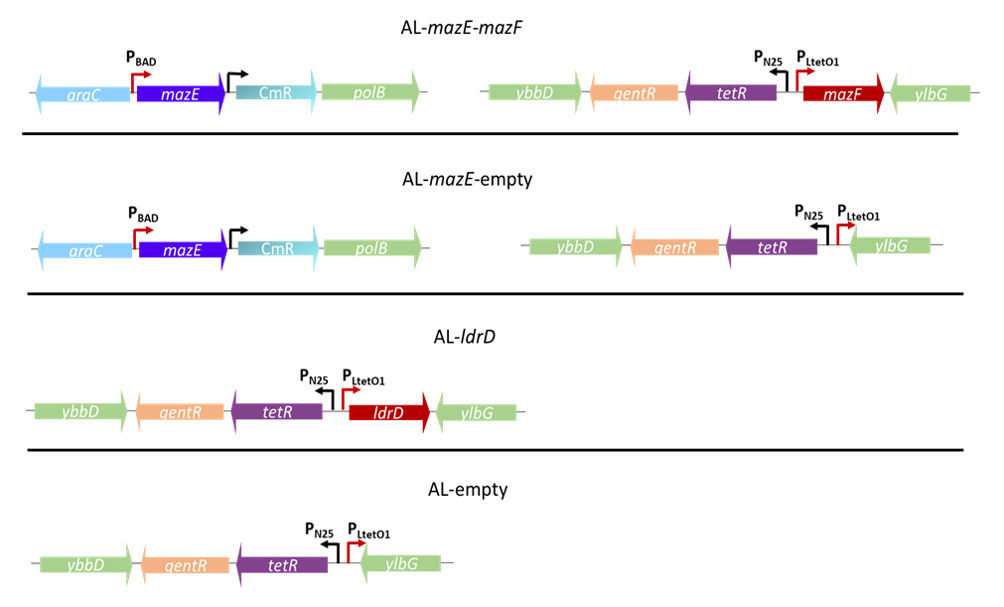

Supplement: FIG S1 [file mbio.01983-21-sf001.tif]

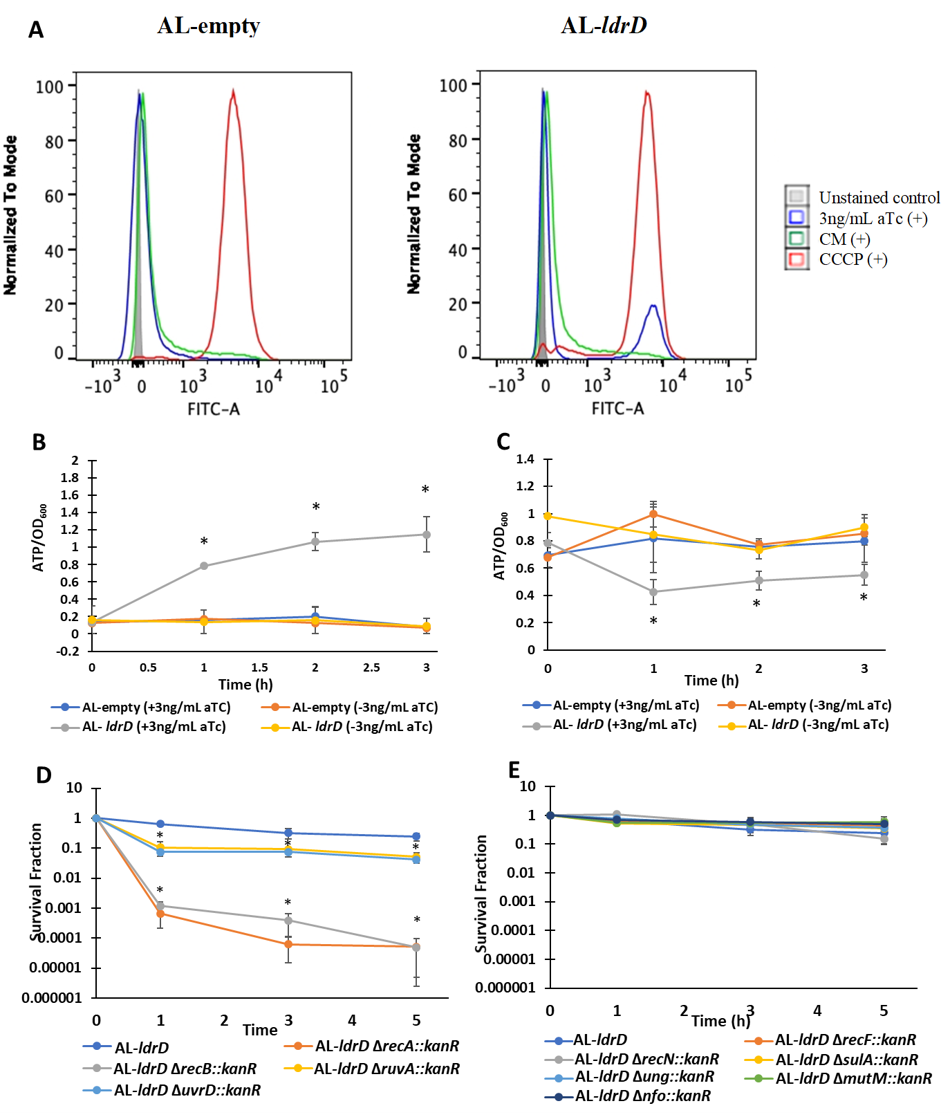

Supplement: FIG S2 [file mbio.01983-21-sf002.tif]

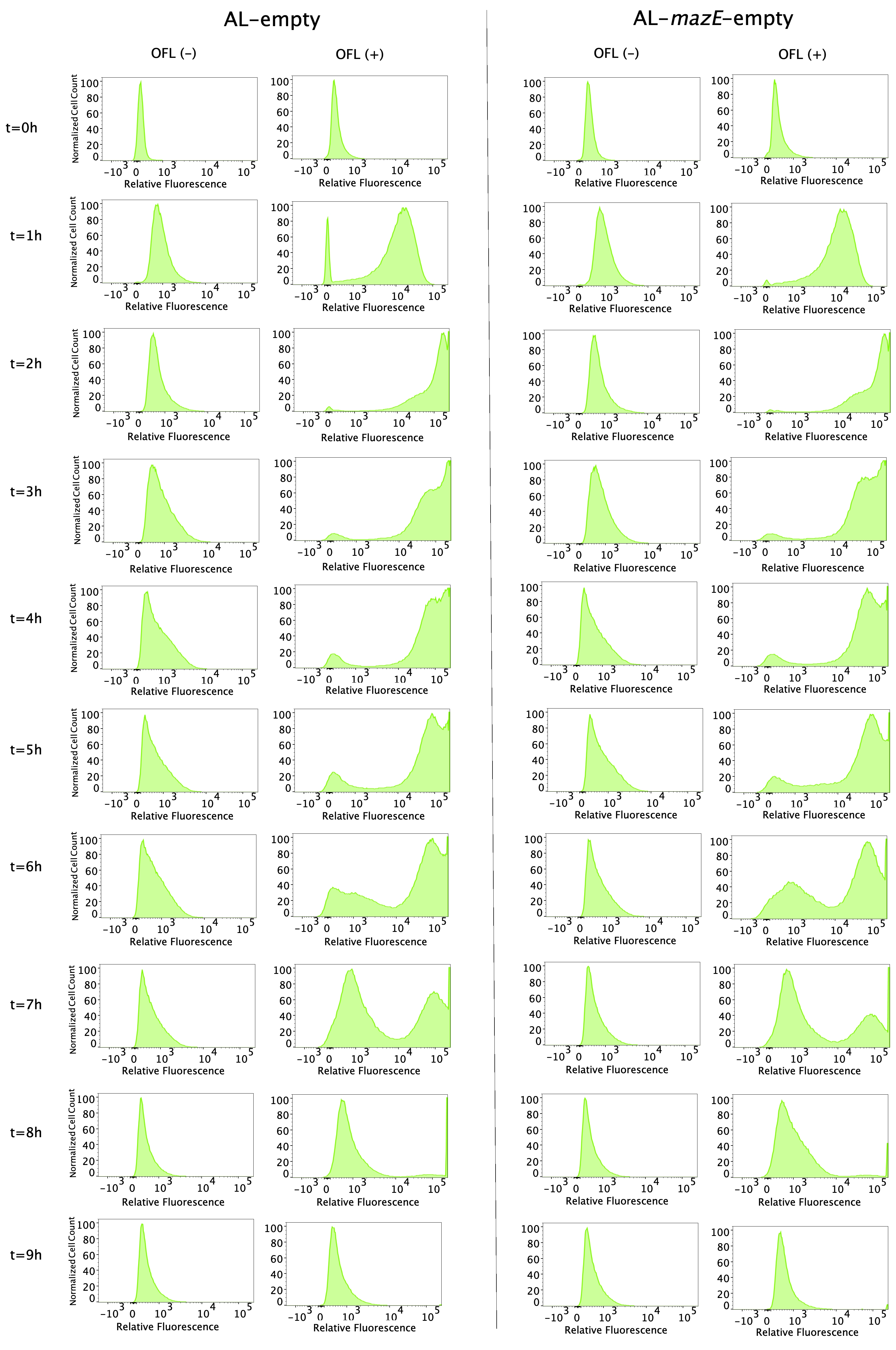

Supplement: FIG S3 [file mbio.01983-21-sf003.tif]

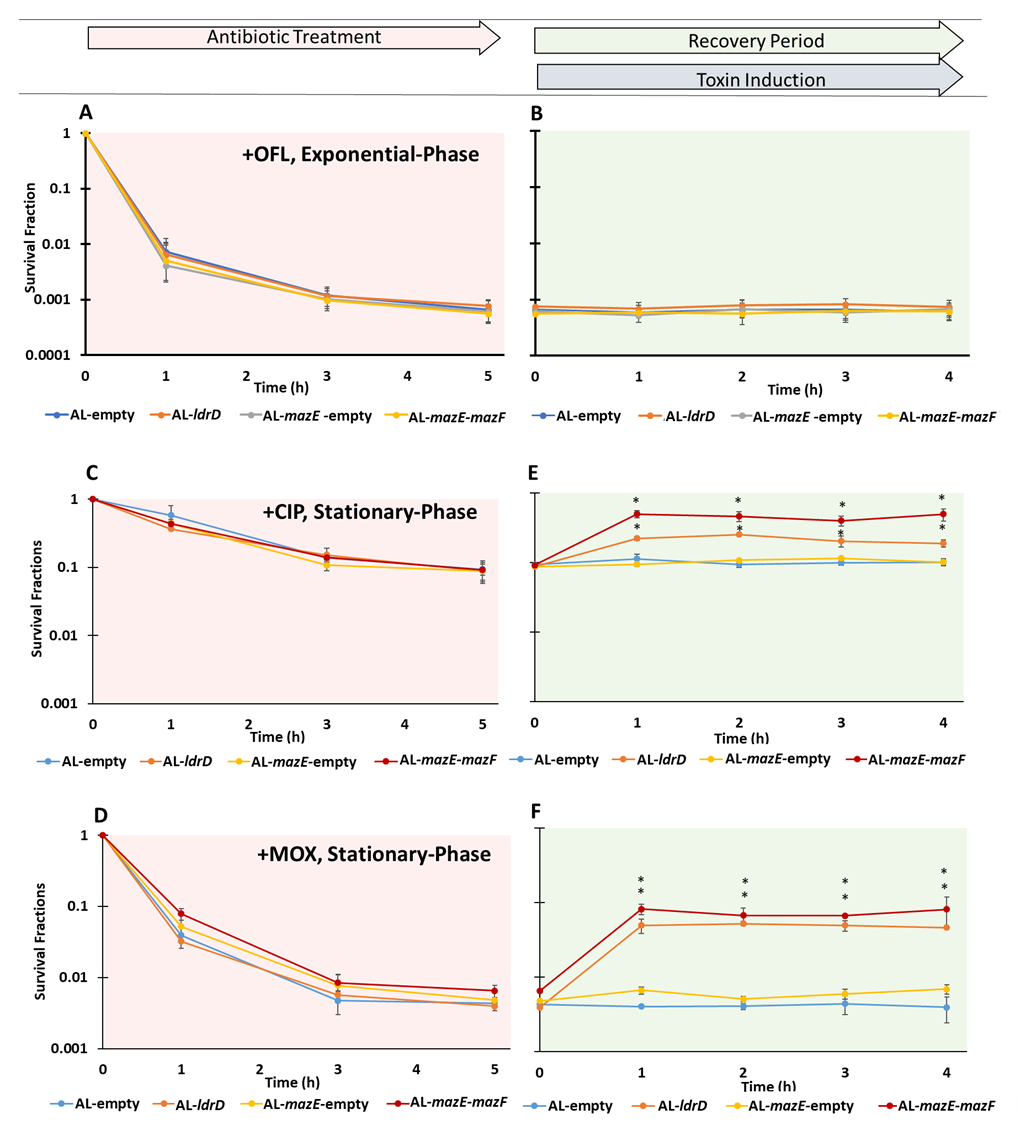

Supplement: FIG S4 [file mbio.01983-21-sf004.tif]

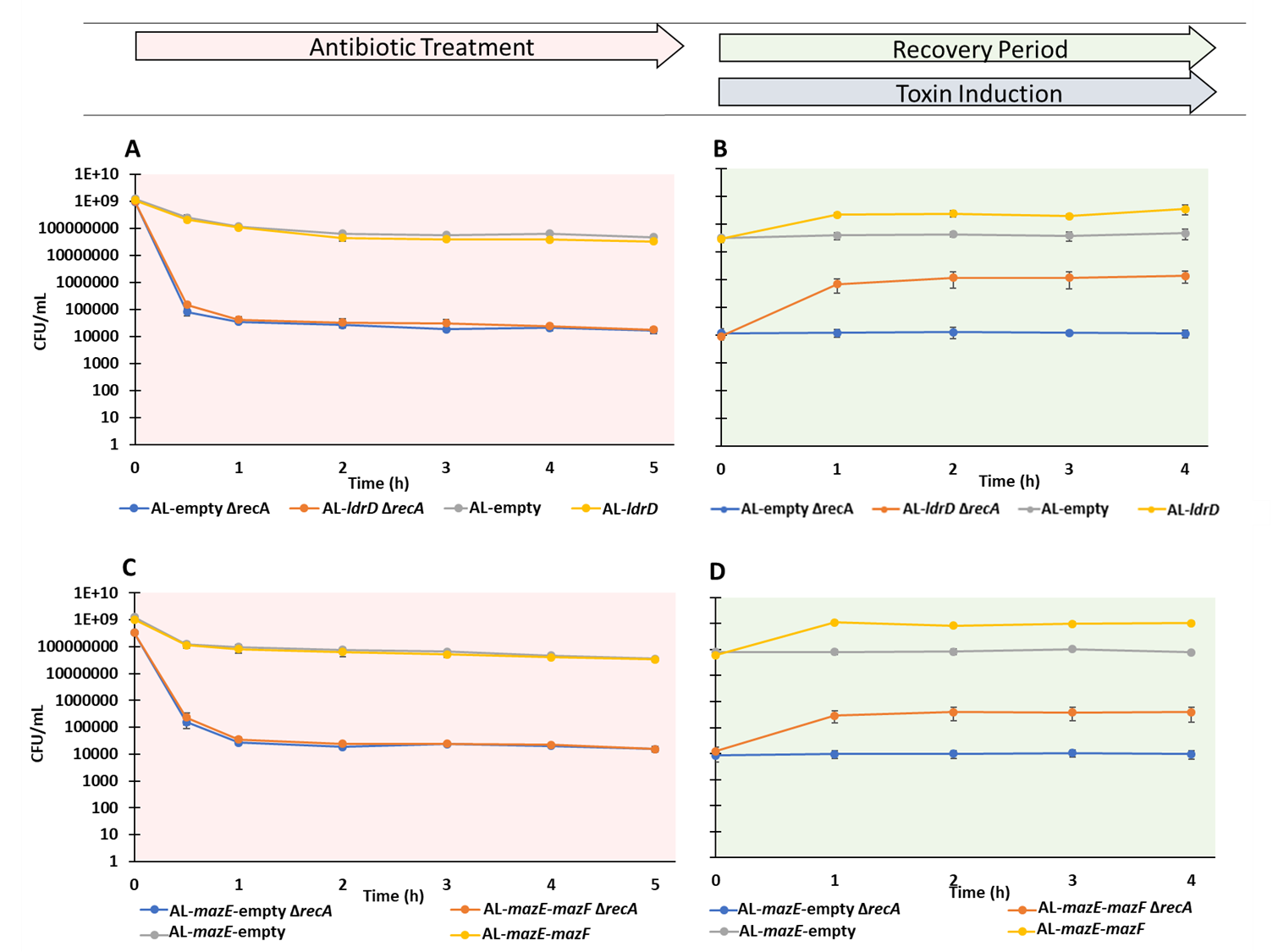

Supplement: FIG S5 [file mbio.01983-21-sf005.tif]

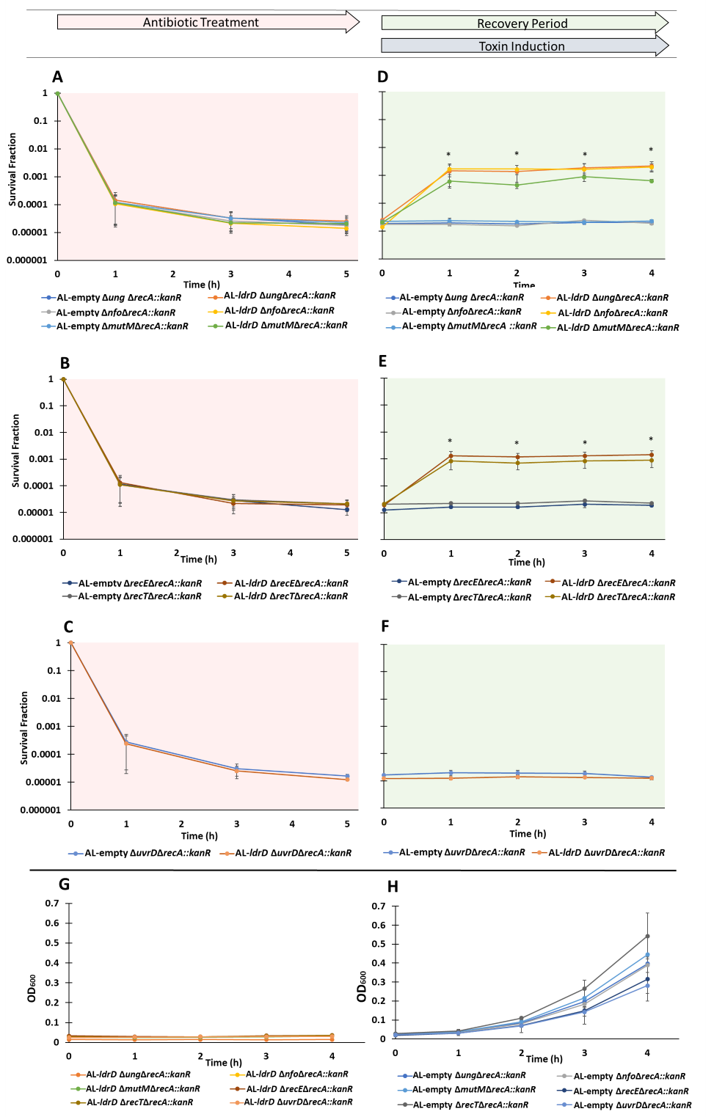

Supplement: FIG S6 [file mbio.01983-21-sf006.tif]

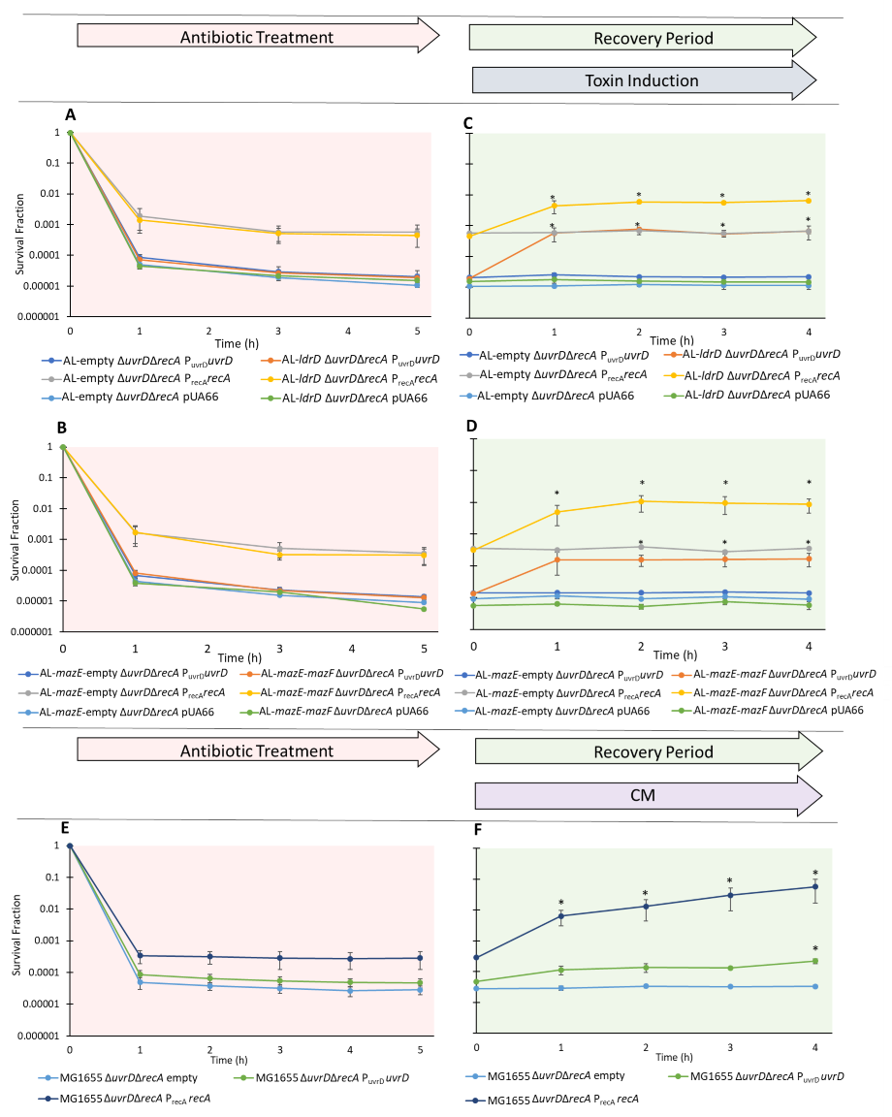

Supplement: FIG S7 [file mbio.01983-21-sf007.tif]

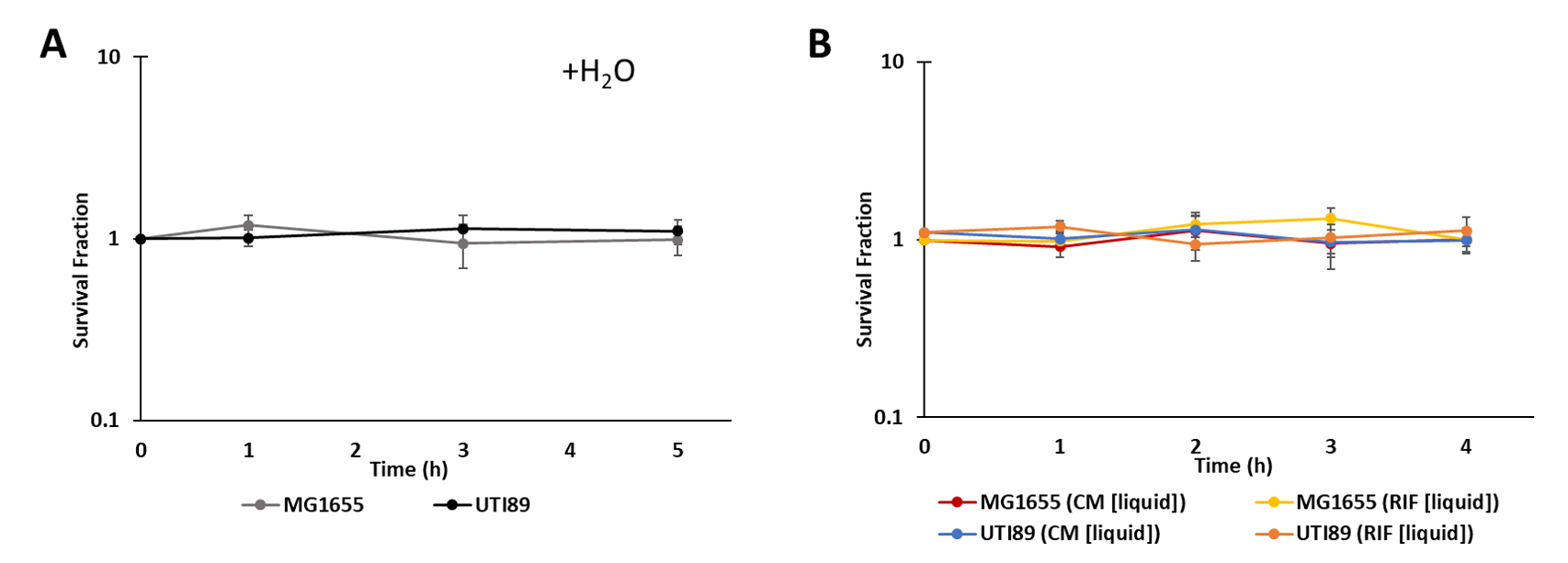

Supplement: FIG S8 [file mbio.01983-21-sf008.tif]
