# Supplementary material for: Toxin Induction or Inhibition of Transcription or Translation Posttreatment Increases Persistence to Fluoroquinolones
Source: mBio. 2021 Aug 17;12(4):e01983-21. doi: 10.1128/mBio.01983-21 (PMC8406316; doi:10.1128/mBio.01983-21)
Supplement: TABLE S1 [file mbio.01983-21-st001.docx]

**Table S1. Bacterial strains and Plasmids**

| **Strains** | | | |
| --- | --- | --- | --- |
| **Strain** | **Relevant genotype** | **Source** | **Description** |
| MG1655 | F-, λ-, *ilvG*-, *rfb-50, rph-1* | ^1^ |  |
| MOcured | MG1655 Δ*lacZYA*::P_lacIQ_*-*P_T5_*-mCherry* | ^2^ |  |
| MOΔ*ldrD/rdlD* | MOcured Δ*ldrD/rdlD* | This work | Deletion of the endogenous *ldrD* and *rdlD* genes |
| AL-empty | MO::Δ*ldrD/rdlD* :: P_LtetO1_ P_N25_-*tetR*-*gent* | This work | Integration of P_LtetO1_, P_N25_-*tetR*, and *gent* between *ybbD* and *ylbG* in MOΔ*ldrD/rdlD* (Fig. S1 in the supplemental material) |
| AL-*ldrD* | MO::Δ*ldrD/rdlD* ::P_LtetO1_-*ldrD* P_N25_-*tetR*-*gent* | This work | Integration of P_LtetO1_-*ldrD*, P_N25_-*tetR*, and *gent* between *ybbD* and *ylbG* in MOΔ*ldrD/rdlD* (Fig. S1 in the supplemental material) |
| MOΔ*mazEF* | MOcured Δ*mazEF* | ^2^ |  |
| MO::*mazE* | MOΔ*mazEF* Δ*araBAD*::P_BAD_-*mazE-cat* | ^2^ |  |
| AL-*mazE*-empty | MO::*mazE*:: P_LtetO1_ P_N25_-*tetR*-*gent* | This work | Integration of P_LtetO1_, P_N25_-*tetR*, and *gent* between *ybbD* and *ylbG* in MO::*mazE* (Fig. S1 in the supplemental material) |
| AL-*mazE*-*mazF* | MO::*mazE*::P_LtetO1_-*mazF* P_N25_-*tetR*-*gent* | ^2^ | (Fig. S1 in the supplemental material) |
| AL-*ldrD* Δ*recA*::*kanR* | AL-*ldrD* Δ*recA*::*kanR* | This work | Deletion of *recA* through P1 transduction of the corresponding deletion mutation from the Keio collection^3^ |
| AL-*ldrD* Δ*recA* | AL-*ldrD* Δ*recA* | This work | Curing AL-*ldrD*:: Δ*recA*::*kanR* of the *kanR* resistance marker using pCP20 |
| AL-*ldrD* Δ*recB*::*kanR* | AL-*ldrD* Δ*recB*::*kanR* | This work | Deletion of *recB* through P1 transduction of the corresponding deletion mutation from the Keio collection^3^ |
| AL-*ldrD* Δ*recF*::*kanR* | AL-*ldrD* Δ*recF*::*kanR* | This work | Deletion of *recF* through P1 transduction of the corresponding deletion mutation from the Keio collection^3^ |
| AL-*ldrD* Δ*recN*::*kanR* | AL-*ldrD* Δ*recN*::*kanR* | This work | Deletion of *recN* through P1 transduction of the corresponding deletion mutation from the Keio collection^3^ |
| AL-*ldrD* Δ*ruvA*::*kanR* | AL-*ldrD* Δ*ruvA*::*kanR* | This work | Deletion of *ruvA* through P1 transduction of the corresponding deletion mutation from the Keio collection^3^ |
| AL-*ldrD* Δ*uvrD*::*kanR* | AL-*ldrD* Δ*uvrD*::*kanR* | This work | Deletion of *uvrD* through P1 transduction of the corresponding deletion mutation from the Keio collection^3^ |
| AL-*ldrD* Δ*uvrD* | AL-*ldrD* Δ*uvrD* | This work | Curing AL-*ldrD*:: Δ*uvrD*::*kanR* of the *kanR* resistance marker using pCP20 |
| AL-*ldrD* Δ*nfo*::*kanR* | AL-*ldrD* Δ*nfo*::*kanR* | This work | Deletion of *nfo* through P1 transduction of the corresponding deletion mutation from the Keio collection^3^ |
| AL-*ldrD* Δ*ung*::*kanR* | AL-*ldrD* Δ*ung*::*kanR* | This work | Deletion of *ung* through P1 transduction of the corresponding deletion mutation from the Keio collection^3^ |
| AL-*ldrD* Δ*mutM*::*kanR* | AL-*ldrD* Δ*mutM*::*kanR* | This work | Deletion of *mutM* through P1 transduction of the corresponding deletion mutation from the Keio collection^3^ |
| AL-*ldrD* Δ*recE* Δ*recA*::*kanR* | AL-*ldrD* Δ*recE*Δ*recA*::*kanR* | This work | Deletion of *recE* through P1 transduction of the corresponding deletion mutation from the Keio collection, followed by curing of the *kanR* resistance marker using pCP20, and subsequent deletion of *recA* through P1 transduction of the corresponding deletion mutation from the Keio collection^3^ |
| AL-*ldrD* Δ*recT* Δ*recA*::*kanR* | AL-*ldrD* Δ*recT*Δ*recA*::*kanR* | This work | Deletion of *recT* through P1 transduction of the corresponding deletion mutation from the Keio collection, followed by curing of the *kanR* resistance marker using pCP20, and subsequent deletion of *recA* through P1 transduction of the corresponding deletion mutation from the Keio collection^3^ |
| AL-*ldrD* Δ*nfo* Δ*recA*::*kanR* | AL-*ldrD* Δ*nfo*Δ*recA*::*kanR* | This work | Curing AL-*ldrD*:: Δ*nfo*::*kanR* of the *kanR* resistance marker using pCP20, followed by deletion of *recA* through P1 transduction of the corresponding deletion mutation from the Keio collection^3^ |
| AL-*ldrD* Δ*ung* Δ*recA*::*kanR* | AL-ldrD Δ*ung*Δ*recA*::*kanR* | This work | Curing AL-*ldrD*:: Δ*ung*::*kanR* of the *kanR* resistance marker using pCP20, followed by deletion of *recA* through P1 transduction of the corresponding deletion mutation from the Keio collection^3^ |
| AL-*ldrD* Δ*mutM* Δ*recA*::*kanR* | AL-*ldrD* Δ*mutM*Δ*recA*::*kanR* | This work | Curing AL-*ldrD*:: Δ*mutM*::*kanR* of the *kanR* resistance marker using pCP20, followed by deletion of *recA* through P1 transduction of the corresponding deletion mutation from the Keio collection^3^ |
| AL-*ldrD* Δ*uvrD*Δ*recA*::*kanR* | AL-*ldrD* Δ*uvrD*Δ*recA*::*kanR* | This work | Curing AL-*ldrD*:: Δ*uvrD*::*kanR* of the *kanR* resistance marker using pCP20, followed by deletion of *recA* through P1 transduction of the corresponding deletion mutation from the Keio collection^3^ |
| AL-*ldrD* Δ*uvrD*Δ*recA* | AL-*ldrD* Δ*uvrD*Δ*recA* | This work | Curing AL-*ldrD*:: Δ*uvrD* Δ*recA*::*kanR* of the *kanR* resistance marker using pCP20 |
| AL-empty Δ*recE*Δ*recA*::*kanR* | AL-empty Δ*recE*Δ*recA*::*kanR* | This work | Deletion of *recE* through P1 transduction of the corresponding deletion mutation from the Keio collection^3^, followed by curing of the *kanR* resistance marker using pCP20, and subsequent deletion of *recA* through P1 transduction of the corresponding deletion mutation from the Keio collection^3^ |
| AL-empty Δ*recT*Δ*recA*::*kanR* | AL-empty Δ*recT*Δ*recA*::*kanR* | This work | Deletion of *recT* through P1 transduction of the corresponding deletion mutation from the Keio collection, followed by curing of the *kanR* resistance marker using pCP20, and subsequent deletion of *recA* through P1 transduction of the corresponding deletion mutation from the Keio collection^3^ |
| AL-empty Δ*nfo*Δ*recA*::*kanR* | AL-empty Δ*nfo*Δ*recA*::*kanR* | This work | Deletion of *nfo* through P1 transduction of the corresponding deletion mutation from the Keio collection, followed by curing of the *kanR* resistance marker using pCP20, and subsequent deletion of *recA* through P1 transduction of the corresponding deletion mutation from the Keio collection^3^ |
| AL-empty Δ*ung*Δ*recA*::*kanR* | AL-empty Δ*ung*Δ*recA*::*kanR* | This work | Deletion of *ung* through P1 transduction of the corresponding deletion mutation from the Keio collection, followed by curing of the *kanR* resistance marker using pCP20, and subsequent deletion of *recA* through P1 transduction of the corresponding deletion mutation from the Keio collection^3^ |
| AL-empty Δ*mutM* Δ*recA*::*kanR* | AL-empty Δ*mutM* Δ*recA*::*kanR* | This work | Deletion of *mutM* through P1 transduction of the corresponding deletion mutation from the Keio collection, followed by curing of the *kanR* resistance marker using pCP20, and subsequent deletion of *recA* through P1 transduction of the corresponding deletion mutation from the Keio collection^3^ |
| AL-empty Δ*uvrD* Δ*recA*::*kanR* | AL-empty Δ*uvrD* Δ*recA*::*kanR* | This work | Deletion of *uvrD* through P1 transduction of the corresponding deletion mutation from the Keio collection, followed by curing of the *kanR* resistance marker using pCP20, and subsequent deletion of *recA* through P1 transduction of the corresponding deletion mutation from the Keio collection^3^ |
| AL-empty Δ*uvrD*Δ*recA* | AL-empty Δ*uvrD*Δ*recA* | This work | Curing AL-empty:: Δ*uvrD* Δ*recA*::*kanR* of the *kanR* resistance marker using pCP20 |
| ΔTOX6 | MG1655::*ΔhokEΔsymEΔtisBΔyafO*Δ*yafQ*Δ*dinQ* | This work | Consecutive deletion of SOS toxins *hokE, symE, tisB* and *yafO* through P1 transduction of the corresponding deletion mutation from the Keio collection^3^ followed by curing of the *kanR* resistance markers with pCP20. *yafQ* and *dinQ* were then deleted using the Datsenko-Wanner method, and each *kanR* resistance marker was again cured using pCP20 |
| AL-*mazE*-*mazF* Δ*uvrD* | AL-*mazE*-*mazF* Δ*uvrD* | This work | Deletion of *uvrD* through P1 transduction of the corresponding deletion mutation from the Keio collection^3^ followed by curing the *kanR* resistance marker using pCP20 |
| AL-*mazE-mazF* Δ*uvrD*Δ*recA* | AL- *mazE-mazF* Δ*uvrD* Δ*recA* | This work | AL-*mazE*-*mazF*:: Δ*uvrD* was cured of the *kanR* resistance marker using pCP20 with subsequent deletion of *recA* through P1 transduction of the corresponding deletion mutation from the Keio collection^3^ followed by curing the *kanR* resistance marker using pCP20 |
| AL-*mazE*-empty Δ*uvrD* | AL-*mazE* Δ*uvrD* | This work | Deletion of *uvrD* through P1 transduction of the corresponding deletion mutation from the Keio collection^3^ followed by curing the *kanR* resistance marker using pCP20 |
| AL-*mazE*-empty Δ*uvrD*Δ*recA* | AL-*mazE*-empty Δ*uvrD*Δ*recA* | This work | Curing of AL-*mazE*-empty:: Δ*uvrD*::*kanR* of the resistance marker with pCP20 followed by deletion of *recA* through P1 transduction of the corresponding deletion mutation from the Keio collection^3^ followed by curing the *kanR* resistance marker using pCP20 |
| MG1655 Δ*uvrD* | MG1655 Δ*uvrD* | This work | Deletion of *uvrD* through P1 transduction of the corresponding deletion mutation from the Keio collection^3^ followed by curing the *kanR* resistance marker using pCP20 |
| MG1655 Δ*uvrD*Δ*recA* | MG1655 Δ*uvrD*Δ*recA* | This work | Curing of MG1655::Δ*uvrD*::*kanR* of its resistance marker with pCP20 followed by deletion of *recA* through P1 transduction of the corresponding deletion mutation from the Keio collection^3^ followed by curing the *kanR* resistance marker using pCP20 |
| AL-*mazE*-empty Δ*recA* | AL- *mazE*-empty Δ*recA* | This work | Deletion of *recA* through P1 transduction of the corresponding deletion mutation from the Keio collection^3^ followed by curing the *kanR* resistance marker using pCP20 |
| AL-*mazE*-*mazF* Δ*recA* | AL- *mazE*- *mazF* Δ*recA* | This work | Deletion of *recA* through P1 transduction of the corresponding deletion mutation from the Keio collection^3^ followed by curing the *kanR* resistance marker using pCP20 |
| UTI89 | Uropathogenic *E.* *coli* (UPEC) | ^4^ | A generous gift from Matthew Mulvey |
| **Plasmids** | | | |
| **Plasmid** | **Relevant genotype** | **Source** | **Description** |
| pUA66 | Vector, pSC101 ori, *kanR*, *gfpmut2* (promoterless) | ^5^ | Empty vector |
| pTOX66 | pUA66 P_LtetO1_- P_N25_-*tetR*-*gent* | ^2^ | AL-empty cloning vector |
| pTOX66-*ldrD* | pUA66 P_LtetO1_-*mazF*-P_N25_-*tetR*-*gent* | This work | AL-*ldrD* cloning vector |
| pUA66-P_recA_-*recA* | pUA66 P_recA_-*recA* | ^6^ | Complementation plasmid |
| pUA66-P_uvrD_-*uvrD* | pUA66 P_uvrD_-*uvrD* | This Work | Complementation plasmid |
| pCP20 | AmpR and CmR, temperature sensitive replication (repA101ts), FLP recombinase gene, FLP | ^7, 8^ | Cloning plasmid for curing FRT-flanked resistance markers |
| pKD46 | AmpR, temperature sensitive replication (repA101ts), encodes λ Red genes (*exo, β, γ*) under P_araB_ promoter | ^8^ | Cloning plasmid used in Datsenko Wanner gene deletion |
| pKD4 | \| KanR, *kanR* expression cassette is flanked by FRT sites \| \| --- \| | ^8^ | Cloning vector used in Datsenko Wanner gene deletion |
| pUA66-P_recA_-*gfp* | pUA66 P_recA_-*gfp* | ^9^ | SOS fluorescent reporter |

**SI REFERENCES**

1. **Kohanski MA, Dwyer DJ, Hayete B, Lawrence CA, Collins JJ.** 2007. A common mechanism of cellular death induced by bactericidal antibiotics. *Cell* **130**:797–810.
2. **Mok WWK, Park JO, Rabinowitz JD, Brynildsen MP.** 2015. RNA Futile Cycling in Model Persisters Derived from MazF Accumulation. *mBio* **6**:e01588-01515.
3. **Baba T, Ara T, Hasegawa M, Takai Y, Okumura Y, Baba M, Datsenko KA, Tomita M, Wanner BL, Mori H.** 2006. Construction of *Escherichia coli* K-12 in-frame, single-gene knockout mutants: the Keio collection. *Mol Syst Biol* **2**:2006.0008.
4. **Debnath I, Norton JP, Barber AE, Ott EM, Dhakal BK, Kulesus RR, Mulvey MA.** 2013. The Cpx stress response system potentiates the fitness and virulence of uropathogenic *Escherichia coli*. *Infect Immun* **81**:1450–1459.
5. **Zaslaver A, Bren A, Ronen M, Itzkovitz S, Kikoin I, Shavit S, Liebermeister W, Surette MG, Alon U.** 2006. A comprehensive library of fluorescent transcriptional reporters for *Escherichia coli.* *Nat Methods* **3**:623–628.
6. **Barrett TC, Mok WWK, Murawski AM, Brynildsen MP**. 2019. Enhanced antibiotic resistance development from fluoroquinolone persisters after a single exposure to antibiotic. *Nat Commun* **10**:1177.
7. **Cherepanov PP, Wackernagel W.** 1995. Gene disruption in *Escherichia coli*: TcR and KmR cassettes with the option of Flp-catalyzed excision of the antibiotic-resistance determinant. *Gene* **158**:9–14.
8. **Datsenko KA, Wanner BL.** 2000. One-step inactivation of chromosomal genes in *Escherichia coli* K-12 using PCR products. *Proc Natl Acad Sci U S A* **97**:6640–6645.
9. **Völzing KG, Brynildsen MP.** 2015. Stationary-Phase Persisters to Ofloxacin Sustain DNA Damage and Require Repair Systems Only during Recovery. *mBio* **6**:e00731-00715.
