# Supplementary material for: Toxin Induction or Inhibition of Transcription or Translation Posttreatment Increases Persistence to Fluoroquinolones
Source: mBio. 2021 Aug 17;12(4):e01983-21. doi: 10.1128/mBio.01983-21 (PMC8406316; doi:10.1128/mBio.01983-21)
Supplement: TABLE S2 [file mbio.01983-21-st002.docx]

**Table S2. DNA oligonucleotides**

| **Primers used in chromosomal perturbations** | | |
| --- | --- | --- |
| **Primer name** | **Sequence** | **Description** |
| dinQ_KO_fwd | AAGCGGATGCATTCTCACTCCATCGCATGGAGAAAACGGGTGTAGGCTGGAGCTGCT | Forward primer used to Wanner *kanR* expression cassette in the place of *dinQ* |
| dinQ_KO_rev | AGAGTAGTGTGCTCTTAGCCCTTAATTACGTTTCCGCTACATATGAATATCCTCCTTA | Reverse primer used to Wanner *kanR* expression cassette in the place of *dinQ* |
| yafQ_KO_fwd | AGACGCCGATGATTTATTTGATAAATTAGGAATTTAAATGTGTAGGCTGGAGCTGCTTCG | Forward primer used to Wanner *kanR* expression cassette in the place of *yafQ* |
| yafQ_KO_rev | CTGTCGGCGCAGGGTTTACAAATTTCATCGGAAGCGGGCTCATATGAATATCCTCCTTA | Reverse primer used to Wanner *kanR* expression cassette in the place of *yafQ* |
| uvrD_XhoI_fwd | CTAGTAGCTCGAGTTACTGCCGCATCTGGAAAT | Forward primer used for constructing complementation plasmid pUA66 P_uvrD_-*uvrD* |
| uvrD_SbfI_rev | TCATCATCCTGCAGGTTACACCGACTCCAGCCGGG | Reverse primer used for constructing complementation plasmid pUA66 P_uvrD_-*uvrD* |
| ylbG_KO_fwd | CTAGTAGCCTGCAGGATGGTCAGCACCGGGCCAGC | Forward primer used to Wanner P_LtetO1_ P_N25_-*tetR*-*gent* or P_LtetO1_*-ldrD* P_N25_-*tetR*-*gent* into the *ylbG-ybbD* region of the genome |
| ybbd_KO_rev | GCAAATAAATTTTTTATGAATCGATTCCCTATCAGTGATAGA | Reverse primer used to Wanner P_LtetO1_ P_N25_-*tetR*-*gent* or P_LtetO1_*-ldrD* P_N25_-*tetR*-*gent* into the *ylbG-ybbD* region of the genome |
| **Primers used to sequence** | | |
| **Primer name** | **Sequence** | **Description** |
| cPCR_ldrD/rdlD_fwd | TCTGGAGTCAGCCCGGATAT | Forward primer external to *ldrD/rdlD* used with *kanR* reverse primer for cPCR check of gene deletion |
| ldrD/rdlD_int_fwd | ATGTTGCGGGGGCTTTATCC | Forward primer internal to *ldrD/rdlD* used for cPCR of *ldrD/rdlD* deletion |
| ldrD/rdlD_int_rev | TGCGGCCTGGTCCGGGCGCA | Reverse primer internal to *ldrD/rdlD* used for cPCR of *ldrD/rdlD* deletion |
| ldrD_Fwd | AAGCCGGAAAGGTTCCGGTG | Forward primer internal to *ldrD* used for cPCR of *ldrD* insertion |
| ldrD_Rev | CTAGTAGGAATTCACGAAAAAGGCCGCAGAGTT | Reverse primer internal to *ldrD* used for cPCR of *ldrD* insertion |
| cPCR_recA_fwd | CTGGTTTGCTTTTGCCACTG | Forward primer external to *recA* used with *kanR* reverse primer for cPCR check of gene deletion; used with cPCR_recA_rev to check for scar sequence |
| cPCR_recA_rev | AATACGCGCAGGTCCATAAC | Reverse primer external to *recA* used with cPCR recA forward to check for deletion of *recA* and scar sequence |
| recA_int_rev | ACGCCTTCGCTATCATCTAC | Reverse primer internal to *recA* used for cPCR of *recA* deletion |
| recA_int_fwd | AAACAAACAGAAAGCGTTGG | Forward primer internal to *recA* used for cPCR of *recA* deletion |
| cPCR_ung_fwd | CATCAACTTATGCGGGTGTG | Forward primer external to *ung* used with *kanR* reverse primer for cPCR check of gene deletion |
| ung_int_fwd | GTCAGGGCGTTCTGCTACTC | Forward primer internal to *ung* used for cPCR of *ung* deletion |
| ung_int_rev | GATGCGGTGCTTTCAGTACA | Reverse primer internal to *ung* used for cPCR of *ung* deletion |
| cPCR_mutM_fwd | GCCAGCACGTGATCTACAAA | Forward primer external to *mutM* used with *kanR* reverse primer for cPCR check of gene deletion |
| mutM_int_fwd | AAGGGCATAATGTGCTGACC | Forward primer internal to *mutM* used for cPCR of *mutM* deletion |
| mutM_int_rev | CAGTGATTCGCTGGCATAGA | Reverse primer internal to *mutM* used for cPCR of *mutM* deletion |
| cPCR_nfo_fwd | AAAGCGTCATCGCATAAACC | Forward primer external to *nfo* used with *kanR* reverse primer for cPCR check of gene deletion |
| nfo_int_for | TTCACCAAAAACCAACGTCA | Forward primer internal to *nfo* used for cPCR of *nfo* deletion |
| nfo_int_rev | GCAACGCTGCATTTCATCTA | Reverse primer internal to *nfo* used for cPCR of *nfo* deletion |
| cPCR_uvrD_fed | TTACTGCCGCATCTGGAAAT | Forward primer external to *uvrD* used with *kanR* reverse primer for cPCR check of gene deletion |
| uvrD_int_fwd | TAATGACAAACAGCGCGAAG | Forward primer internal to *uvrD* used for cPCR of *uvrD* deletion |
| uvrD_int_rev | CAGACGCCCGTTATTGTTTT | Reverse primer internal to *uvrD* used for cPCR of *uvrD* deletion |
| cPCR_recB_fwd | AACGGGAAAGCCGAATATGTACAC | Forward primer external to *recB* used with *kanR* reverse primer for cPCR check of gene deletion |
| recB_int_fwd | GCGGAAGATCTGCGTTTGCT | Forward primer internal to *recB* used for cPCR of *recB* deletion |
| recB_int_rev | TCATAGCGGTGTGCCTGCAT | Reverse primer internal to *recB* used for cPCR of *recB* deletion |
| cPCR_ruvA_fwd | CATCGAGACACCTCGCAAGTT | Forward primer external to *ruvA* used with *kanR* reverse primer for cPCR check of gene deletion |
| ruvA_int_fwd | AAGTGGGCGGCGTAGGCTAT | Forward primer internal to *ruvA* used for cPCR of *ruvA* deletion |
| ruvA_int_rev | GCGGCTTGCTTCTTGTGGTT | Reverse primer internal to *ruvA* used for cPCR of *ruvA* deletion |
| cPCR_recF_fwd | GCGAAAACGTCCGCATGATG | Forward primer external to *recF* used with *kanR* reverse primer for cPCR check of gene deletion |
| recF_int_fwd | TCCCTCACCCGCTTGTTGAT | Forward primer internal to *recF* used for cPCR of *recF* deletion |
| recF_int_rev | TGATCGCGCTGACAAAGACC | Reverse primer internal to *recF* used for cPCR of *recF* deletion |
| cPCR_yafO_fwd | CCGGTTGCGGTTCTTTCTAA | Forward primer external to *yafO* used with *kanR* reverse primer for cPCR check of gene deletion |
| yafO_int_fwd | TAAGGATGCGGGTATTCAAA | Forward primer internal to *yafO* used for cPCR of *yafO* deletion |
| yafO_int_rev | CGCCAGGCTGATAGTTTCTTA | Reverse primer internal to *yafO* used for cPCR of *yafO* deletion |
| cPCR_yafQ_fwd | TGGTTCGCATAACCCTCACA | Forward primer external to *yafQ* used with *kanR* reverse primer for cPCR check of gene deletion |
| yafQ_int_fwd | TGAATACTCGGGACAATATTCAAA | Forward primer internal to *yafQ* used for cPCR of *yafQ* deletion |
| yafQ_int_rev | GGAAGGCTCACATTATCACCAA | Reverse primer internal to *yafQ* used for cPCR of *yafQ* deletion |
| cPCR_tisAB_fwd | TCACAGTTTGCGTTTTGTCC | Forward primer external to *tisAB* used with *kanR* reverse primer for cPCR check of gene deletion |
| tisAB_int_fwd | AAGCACAACGTTTCTCCTTGAG | Forward primer internal to *tisAB* used for cPCR of *tisAB* deletion |
| tisAB_int_rev | GCGGGAGAGGAATCTATCAC | Reverse primer internal to *tisAB* used for cPCR of *tisAB* deletion |
| symE_int_fwd | ACTGACACGCATTCTATTGCAC | Forward primer internal to *symE* used for cPCR of *symE* deletion |
| symE_int_rev | GCTGTTACGCGACTTTCTGTT | Reverse primer internal to *symE* used for cPCR of *symE* deletion |
| cPCR_hokE_fwd | GGAGTGAGAAGTCCGAAACAGG | Forward primer external to *hokE* used with *kanR* reverse primer for cPCR check of gene deletion |
| hokE_int_fwd | CGGCAGTCATAGTGCTGTGT | Forward primer internal to *hokE* used for cPCR of *hokE* deletion |
| hokE_int_rev | TTCGGTTCGTAAGCGAGAAC | Reverse primer internal to *hokE* used for cPCR of *hokE* deletion |
| cPCR_recE _fwd | AGCCACTGTTGTAACTACCGA | Forward primer external to *recE* used with *kanR* reverse primer for cPCR check of gene deletion of *recE* and *recT* |
| cpcR_recE_rev | AATCGACCTGTGCCACTCTG | Reverse primer external to *recE* used with cPCR recE forward to check for deletion of recE gene and scar sequence |
| recE_int_fwd | AATCGACCTGTGCCACTCTG | Forward primer internal to *recE* used for cPCR of *recE* deletion |
| recE_int_rev | CCTCGATCGGTGTAGTCAGC | Reverse primer internal to *recE* used for cPCR of *recE* deletion |
| recT_int_fwd | ATGATCCGTATCGCCACCAC | Forward primer internal to *recT* used for cPCR of *recT* deletion |
| recT_int_rev | GGCGACGAATAGCCGTTTTC | Reverse primer internal to *recT* used for cPCR of *recT* deletion |
| KanR_rev | TGTAGATCGCTGAACTTGTAGGCCTGATAAGCGCAGCGTATCAGGCAATTTCAGAAGAACTCGTCAAGAAGG | Reverse primer internal to *kanR* resistance marker used to check the insertion of the *kanR* resistance marker after P1 transduction or Datsenko-Wanner method |
